# Latitude distribution characteristics of soil microbial communities in the *Ziziphus jujuba* var. *spinosa* shrublands on the western slope of Taihang Mountains

**DOI:** 10.3389/fmicb.2026.1729146

**Published:** 2026-04-10

**Authors:** Xin Zhang, Yaxuan Chen, Mengyu Li, Yanmei Chen, Jingze Liu

**Affiliations:** 1 Hebei Key Laboratory of Animal Physiology, Biochemistry and Molecular Biology, Hebei Collaborative Innovation Center for Eco-Environment, College of Life Sciences, Hebei Normal University, Shijiazhuang, China; 2 School of Geographical Sciences, Hebei Key Laboratory of Environmental Change and Ecological Construction, Hebei Normal University, Shijiazhuang, China

**Keywords:** microbial community diversity, microbial composition, latitude variation, Taihang Mountains, *Ziziphus jujuba* var. spinosa shrublands

## Abstract

**Introduction:**

Investigating soil microbial spatial distribution is essential for understanding ecosystem responses to climate change. Studies on single vegetation types and their influencing factors are limited.

**Methods:**

We focused on soil samples from *Ziziphus jujuba* var. *spinosa* shrublands in the western foothills of the Taihang Mountains. High-throughput sequencing and bioinformatic techniques were used to analyze the diversity and structure of microbial communities. At the same time, soil physicochemical properties and vegetation characteristics were measured to identify the factors driving microbial community variation.

**Results:**

The results showed that dominant bacterial and fungal groups generally exhibited a “unimodal” or “U-shaped” distribution pattern along the latitudinal gradient, with both the highest and lowest values occurring in the mid-section of the study area. Bacterial alpha diversity was higher than fungal alpha diversity. Along the latitudinal gradient, bacterial alpha diversity followed a nonlinear cubic trend, peaking in the southern section and reaching a minimum in the northern section. For fungi, richness displayed a U-shaped pattern, Shannon and evenness indices increased linearly, and the Simpson index decreased linearly. Pearson correlation and redundancy analyses indicated that soil total nitrogen, sand content, total carbon, total phosphorus, litter carbon, and leaf water content influenced bacterial community composition. Fungal communities were primarily affected by mean annual precipitation, soil total nitrogen, nitrate nitrogen, silt content, leaf water content, and litter nitrogen.

**Discussion:**

These findings highlight the complex spatial patterns of soil microbial communities in shrublands and reveal key environmental drivers shaping their distribution. Understanding these patterns provides a critical basis for predicting how soil microbial diversity and composition may respond to environmental changes along latitudinal gradients.

## 1 Introduction

Soil microorganisms are a vital component of soil ecosystems. They participate in critical ecological processes, including material cycling and energy flow (Wagg et al., 2014; Chen et al., 2020; Zhang et al., 2022). These processes play a key role in maintaining the dynamic balance of soil ecosystems. These microorganisms drive biogeochemical cycles and the turnover of soil organic matter. They also improve soil fertility and quality and are linked to plant productivity (Zelles, 1999). During plant growth, soil microorganisms contribute to soil structure formation and organic matter decomposition. They also facilitate mineral breakdown, nitrogen fixation, regulate plant growth, and protect plants against soil-borne diseases (Li and Hu, 2000). Studying the composition and diversity of soil microbial communities is crucial. It helps improve soil ecological environments and promotes the sustainable use of soil resources (Huang et al., 2018; Chu and Shen, 2017).

Numerous studies have confirmed that the composition and diversity of soil microbial communities vary significantly across spatial scales (He and Wang, 2015; Bardgett and van der Putten, 2014; Yang et al., 2021). Latitude is one of the primary factors influencing the composition and diversity of microbial communities (Fierer and Lennon, 2011). Researchers have reported different patterns in the latitudinal distribution of microbial communities. For instance, Duan et al. (2022) found that the *α*-diversity of bacteria and eukaryotes in the desert regions of the Hexi Corridor in northern China decreased with increasing latitude. Bahram et al. (2018) and van den Hoogen et al. (2019) investigated diversity trends from polar to tropical regions. They found that soil bacterial diversity was highest at mid-latitudes. Fungal diversity gradually decreased with increasing latitude. Teng et al. (2021) studied nine forest ecosystems in eastern China. They reported that saprotrophic and pathogenic fungi were most abundant in tropical and subtropical forests. Their abundance increased with latitude, peaking in temperate forests. Xiao et al. (2021) found that in paddy field ecosystems, soil microbial β diversity decreased significantly with latitude. The most pronounced declines occurred in the β diversity of archaea (Woesearchaeota), bacteria (Bacteroidota), and functional genes related to methane production. However, other studies have indicated that soil bacterial diversity in the Northern Hemisphere does not exhibit a declining trend with increasing latitude. Although microbial community composition varies with latitude, the distribution patterns of soil microbial relative abundance remain unclear (Fierer and Jackson, 2006; Chu et al., 2010; Wu et al., 2011; Yu et al., 2022; Huang et al., 2024; Zhou et al., 2024). O’Malley (2007) suggested that microbial communities are distributed globally in a random manner, without distinct zonal or regional distribution characteristics. Compared to macroorganisms, research on the spatial distribution of soil microbial communities has not yet reached a consensus. Further studies on the spatial distribution of soil microbial communities are urgently needed. These studies will provide a scientific basis for a systematic understanding of this topic.

The composition and diversity of soil microbial communities are important indicators of soil quality and health. They respond sensitively to changes in environmental factors (Vieira et al., 2020). Soil microbial communities are also influenced by precipitation and temperature (Liu et al., 2014), soil pH (Brockett et al., 2012; Tripathi et al., 2012; Semenov et al., 2018; Gao et al., 2018), and soil nutrients (Ramirez et al., 2012; Lauber et al., 2008; Cui et al., 2018). Plants influence soil microbial diversity and composition by modifying soil physicochemical properties (He and Wang, 2015). Microbial communities with high richness and stable structure can fully exert their ecological functions, thereby improving plant productivity (Oldroyd and Leyser, 2020). With advances in microbial molecular technology, research on soil microorganisms in various habitats and vegetation types has increased (Du et al., 2024; Chodak et al., 2016; Kang et al., 2023; Fu et al., 2020). However, limited research has examined variations in the abundance and structure of soil microbial communities within the same climatic zone and vegetation type along latitudinal gradients. Studies on soil microbial communities in *Ziziphus jujuba* var. *spinosa (Z. jujuba)* shrublands across latitudes are even scarcer. Latitudinal variations drive changes in environmental factors, which can influence the composition and structure of soil microbial communities. However, the primary factors responsible for these changes remain unclear. Therefore, this study aims to better understand how soil microorganisms respond to latitudinal changes and environmental factors in wild vegetation. It also seeks to provide new insights into the latitudinal distribution patterns and influencing factors of soil microbial communities.

It also seeks to provide new insights into the latitudinal distribution patterns and influencing factors of soil microbial communities. *Z. jujuba* shrublands perform critical ecological functions in arid, semi-arid, and ecologically fragile zones. These functions include soil and water conservation, windbreak and sand fixation, biodiversity maintenance, and soil improvement. The presence of *Z. jujuba* significantly influences the structure, diversity, and functional activity of soil microorganisms. This, in turn, regulates key soil ecological processes. Therefore, we collected soil samples from *Z. jujuba* shrublands naturally distributed along different latitudinal gradients in the western foothills of the Taihang Mountains. We used high-throughput sequencing and soil physicochemical analyses to characterize soil microbial communities and measure various environmental factors.

We aim to address the following questions: (1) What is the distribution pattern of soil microbial community composition in *Z. jujuba* shrublands along different latitudinal gradients? (2) How do soil microbial community diversity and structure vary along these gradients? (3) What are the main environmental factors driving changes in microbial communities? The results can reveal the spatial distribution patterns of microbial communities in the Taihang Mountains and their environmental adaptation mechanisms. They provide a scientific basis for predicting the impacts of climate change on ecosystems and for promoting the sustainable development of the Taihang Mountains ecosystem.

## 2 Study sites and research methods

### 2.1 Study sites

The Taihang Mountains are an important geographical boundary and physiographic landmark in eastern China. They span approximately 400 kilometers from north to south across Beijing, Hebei, Shanxi, and Henan provinces. This mountain range forms a natural divide between the Loess Plateau and the North China Plain. The study area is located along the western foothills of the Taihang Mountains (37°24′53.55”N–40°9′4.59”N, 114°1′14.89″E–115°8′50.66″E). It extends from south to north through Yuncheng, Jincheng, Linfen, Changzhi, Jinzhong, Taiyuan, Xinzhou, and Datong in Shanxi Province, and Zhangjiakou in Hebei Province. Characterized by a warm-temperate continental monsoon climate, the region exhibits distinct seasonal variations with moderate precipitation. The region is hot and humid in summer and cold and dry in winter. The mean annual temperature ranges from −5 °C to 23 °C. The average annual precipitation is approximately 534 mm, showing notable hydrothermal gradient variations. The predominant soil types are cinnamon soil and leached cinnamon soil. The main tree species include *Ziziphus jujuba* var. *spinosa*, *Vitex negundo var. heterophylla*, *Broussonetia papyrifera*, *Ulmus pumila*, and *Lespedeza bicolor* (Supplementary Appendix Figure 1).

### 2.2 Sample plot setting and collection

Ten natural habitat sites of *Z. jujuba* shrublands along the Taihang Mountains, from south to north, were randomly selected. At each site, five soil samples were collected and combined into one composite sample using the five-point sampling method. Plant samples, including *Z. jujuba* leaves and surface litter, were collected from the shrubs corresponding to the soil samples. Five replicates were set up for each plot. Terrain factors, including latitude, longitude, altitude, slope, and aspect, were recorded for each sampling point using a GPS system. XX, YC, and GX were designated as the southern section of the Taihang Mountains. QX, TG, SY, and YQ were designated as the central section. DX, LQ, and ZL were designated as the northern section. The basic information of the sampling sites is shown in Supplementary Appendix Figure 1 and Table 1.

**Table 1:** Basic information of the study area.

Sites		Longitude and latitude	Altitude/m	Mean annual temperature/°C	Mean annual precipitation/mm	Aspect	Soil types	Dominant and accompanying vegetation
Southern section of Taihang Mountains	XX	35° 4′19.03″N, 111° 12′28.31″E	511	12.43	659.17	Southwest	Leached brown soil	*Ziziphus jujuba* var. *spinosa*, *Broussone-tia papyrifera* (L.) Vent., *Prunus persica* (L.) Batsch, *Platycladus orientalis*, *Robinia pseudoacacia* L.
	YC	35° 29′34.41″N, 112° 35′4.04″E	695	13.81	781.3 7	Southwest	Leached brown soil	*Ziziphus jujuba* var. *spinosa*, *Ulmus pumila* L., *Broussone-tia papyrifera* (L.) Vent., *Platycladus orientalis*, *Citrus reticulata* Blanco
	GX	36° 11′21.78″N, 111° 57′29.33″E	786	12.34	521.49	Southwest	Leached brown soil	*Ziziphus jujuba* var. *spinosa*, *Ulmus pumila* L.*, Prunus armeniaca* L.*, Lespedeza bicolor* Turcz.
Middle section of Taihang Mountains	QX	36° 42′12.30″N, 112° 42′28.58″E	962	11.28	516.73	Southeast	Leached brown soil	*Ziziphus jujuba* var. *spinosa*, *Ulmus pumila* L., *Artemisia annua* L., *Leonurus japonicus* Houtt.
	TG	37° 25′5.00″N, 112° 42′50.52″E	866	12.03	416.84	Southwest	Cinnamon soil	*Ziziphus jujuba* var. *spinosa*, Jujubae Fructus, *Ulmus pumila* L., *Salsola collina* Pall. P*ortulaca oleracea* L., *Bidens pilosa* L.
	SY	37° 48′50.02″N, 113° 8′50.24″E	1,036	10.32	403.21	Southwest	Leached brown soil	*Ziziphus jujuba* var. *spinosa*, *Ulmus pumila* L.
	YQ	38° 4′39.78″N, 112° 40′30.80″E	901	11.58	408.08	Southwest	Cinnamon soil	*Ziziphus jujuba* var. *spinosa*, *Ailanthus altissima* (Mill.) Swingle, *Ulmus pumila* L.
North section of Taihang Mountains	DX	39° 5′56.38″N, 112° 52′37.47″E	1,007	10.62	475.14	Southeast	Cinnamon soil	*Ziziphus jujuba* var. *spinosa*, *Lespedeza davurica* (Laxm.) Schindl., *Setaira viridis*(L.)Beauv
	LQ	39° 24′2.60″N, 114° 17′19.82″E	912	8.83	541.53	Southwest	Cinnamon soil	*Ziziphus jujuba* var. *spinosa*, *Ulmus pumila* L., *Periploca sepium*,
	ZL	40° 9′4.59″N, 115° 8′50.66″E	1,052	7.35	510.78	South	Cinnamon soil	*Ziziphus jujuba* var. *spinosa*, *Ulmus pumila* L., *Artemisia mongolica* Fisch.

XX refers to Xia County, Yuncheng City, Shanxi Province; YC refers to Yangcheng County, Jincheng City, Shanxi Province; GX refers to Gu County, Linfen City, Shanxi Province; QX refers to Qin County, Changzhi City, Shanxi Province; TG refers to Taigu County, Jinzhong City, Shanxi Province; SY refers to Shouyang County, Jinzhong City, Shanxi Province; YQ refers to Yangqu County, Taiyuan City, Shanxi Province; DX refers to Daixian County, Xinzhou City, Shanxi Province; LQ refers to Lingqiu County, Datong City, Shanxi Province; ZL refers to Zhuolu County, Shijiazhuang City, Hebei Province.

Specific collection method: Soil samples were collected using the five-point sampling method. Soil was excavated within 20 cm of the plant roots at a depth of 10–20 cm. Approximately 100 g of soil was collected at each sampling point. Soil samples were collected from 4–5 trees and combined to form one composite replicate. Five replicates were established for each plot. In total, 50 soil samples, 50 leaf samples, and 50 litter samples were collected from 10 plots. After labeling, the soil samples were immediately placed in an ice box and transported to the laboratory in a portable refrigerator set at −20 °C (Ice Tiger Alpine, Foshan Aikai Electric Appliance Co., Ltd., China). Approximately 10 g of fresh soil was subsampled for DNA extraction and stored at −80 °C (DW-86 L486, Haier Biomedical Co., Ltd., Qingdao, Shandong, China). Another portion was sieved through a 2 mm mesh and stored at 4 °C for the analysis of soil available nutrients. The remaining soil was air-dried at room temperature. Visible roots, plant debris, and animal residues were removed. The dried soil was sieved and stored at 25 ± 2 °C for physicochemical analysis. The fresh weights of plant leaves and litter were measured. The samples were oven-dried, ground into powder, and passed through a 100-mesh sieve for nutrient content analysis.

### 2.3 Determination of environmental factor indicators

Fresh soil collected using a cutting ring was dried at 105 °C to constant weight for determination of soil bulk density (BD) and soil water content (SWC). Soil total nitrogen (TN) was measured using the Kjeldahl method (NY/T 1121.24–2012). Ministry of Agriculture of the People’s Republic of China (2012). Soil total carbon (TC) was determined by combustion–infrared absorption spectroscopy (Chen et al., 2023). Soil organic carbon (SOC) was analyzed using the potassium dichromate external heating method (NY/T 1121.6–2006) Ministry of Agriculture of the People’s Republic of China (2006). Soil total phosphorus (TP) was determined by sodium hydroxide fusion followed by molybdenum–antimony colorimetry (UV-1801C, Beijing Beifenluoli Analytical Instrument Co., Ltd.)(LY/T 1232–2015) National Forestry Administration of China (2015). Soil pH and electrical conductivity (EC) were measured in a 5:1 (water:soil, v/m) suspension using a multiparameter analyzer (Wang, 2022). Soil particle size distribution was determined according to NY/T 1121.3–2006, Ministry of Agriculture of the People’s Republic of China (2006), using a laser particle size analyzer, and the proportions of sand (0.02–2.0 mm), silt (0.002–0.02 mm), and clay (< 0.002 mm) were calculated. Soil ammonium and nitrate nitrogen were quantified using a commercial assay kit with absorbance measured by a microplate reader (Multiskan GO, Thermo Fisher Scientific, USA). Plant total carbon was determined using a combustion–nonaqueous titration method (DZ/T 0279.26–2016). Ministry of Land and Resources of the People’s Republic of China (2016). Plant total nitrogen was measured using the Nessler colorimetric method with UV spectrophotometry (UV-1801C, Beijing Beifenluoli Analytical Instrument Co., Ltd.)(Sun, 2011).

### 2.4 Determination of soil microbial community characteristics

Refer to the soil DNA extraction kit (FastDNA spin kit for soil, MP Biomedicines, Cleveland, USA) to have total soil DNA extracted from 0.5 g of soil. The integrity of the extracted DNA was detected by 1% agarose gel electrophoresis. Primers 338F (5’-ACTCCTACGGGAGGCAGCAG-3′) and 806R (5′- GGACTACHVGGGTWTCTAAT-3′) were selected to amplify the V3 and V4 regions of bacterial 16S rRNA. Primers 1737F (5 ‘- GGAAGTAAAAGTCGTAACAAGG −3′) and 2043R (5 ‘- GCTGCGTTCTTCATCGATGC -3′) were selected to amplify the internal transcribed spacer (ITS) region of fungi. PCR amplification was performed to synthesize specific products with barcodes, which were mixed evenly according to their concentration. They were then detected using 2% agarose gel electrophoresis. The PCR products were gel-extracted using the AxyPrep DNA gel extraction kit (AXYGEN company), and the extracted products were quantified using the QuantiFluor™-ST blue fluorescence quantification system (Promega company). The library was constructed using the TruSeqTM DNA Sample Prep Kit. After passing the quality control, sequencing analysis was performed using the Illumina® MiSeq sequencer (Illumina, San Diego, USA). After sequencing, UPARSE software was used to cluster operational taxonomic units (OTUs). Each OTU was then normalized and subjected to species annotation and classification (Shanghai Majorbio Technology Co., Ltd.).

### 2.5 Statistical analysis

Basic statistical parameters were calculated using Excel 2019. One-way ANOVA was performed using SPSS 26 to test differences among environmental factors. Alpha diversity indices were calculated in mothur (v1.30.2) at a 97% OTU similarity threshold. Microbial community diversity metrics were obtained using QIIME. Principal coordinates analysis (PCoA) based on Bray–Curtis distances was performed using the “vegan” package in R to assess beta diversity and community structure. Permutational multivariate analysis of variance (PERMANOVA) was conducted to evaluate the effects of grouping factors on microbial community variation. To determine the relative contributions of deterministic and stochastic processes to community assembly, a null model with 999 randomizations was used to calculate βNTI. When |βNTI| ≥ 2, deterministic processes were considered dominant; when |βNTI| < 2, stochastic processes were considered dominant (Wang et al., 2024). A neutral community model was further applied to examine species abundance distributions (Huang et al., 2025). Redundancy analysis (RDA) was performed using Canoco 5 and the “vegan” package in R to explore the relationships between vegetation, soil properties, and microbial communities. Spearman correlation analysis was conducted to assess pairwise relationships among variables. All multivariate analyses were performed on the Meiji Bio Cloud platform based on R 4.3.0. Figures were generated using Origin 2021, GraphPad Prism 8.0, and Chiplot.

## 3 Results and analysis

### 3.1 Latitudinal variation in soil microbial community composition

**Figure 1:**
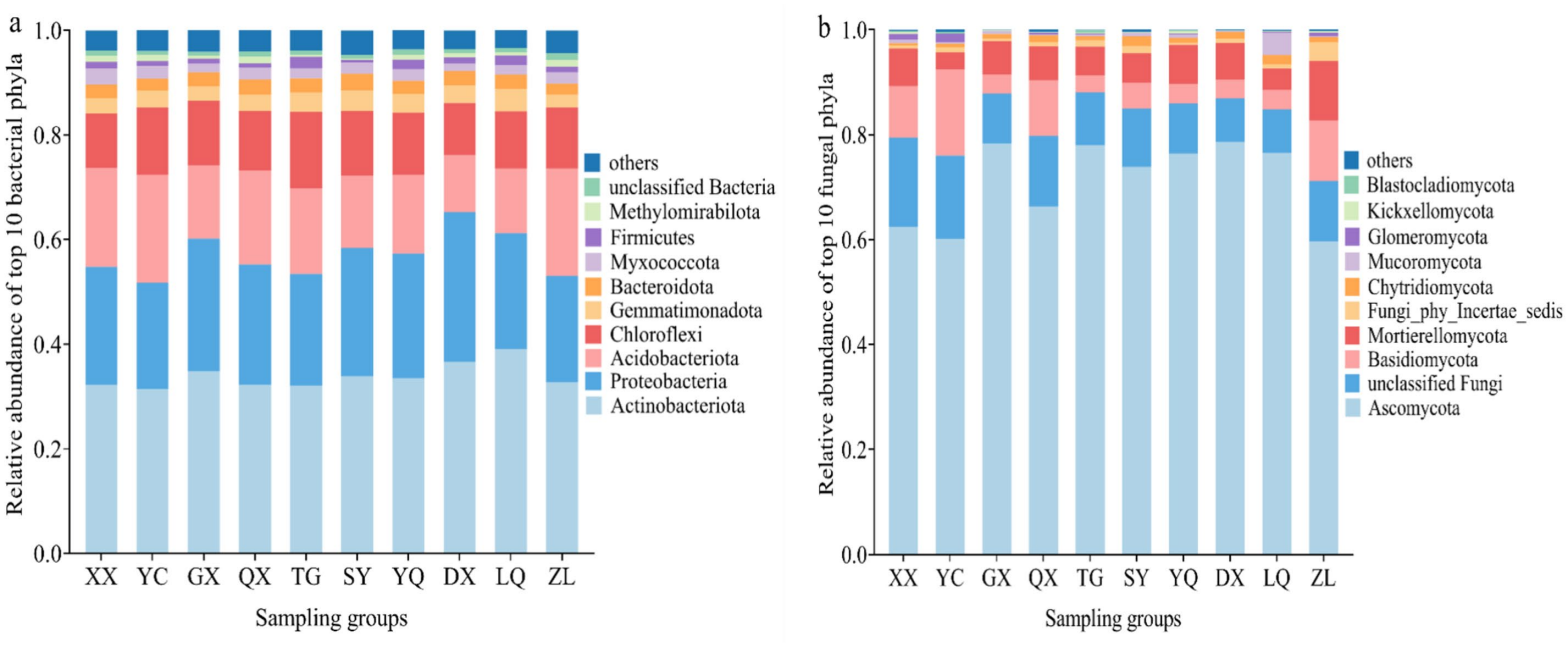
Top 10 soil microorganisms in terms of relative abundance at the phylum level: **(a)** Bacterial phyla; **(b)** Fungal phyla.

**Figure 2:**
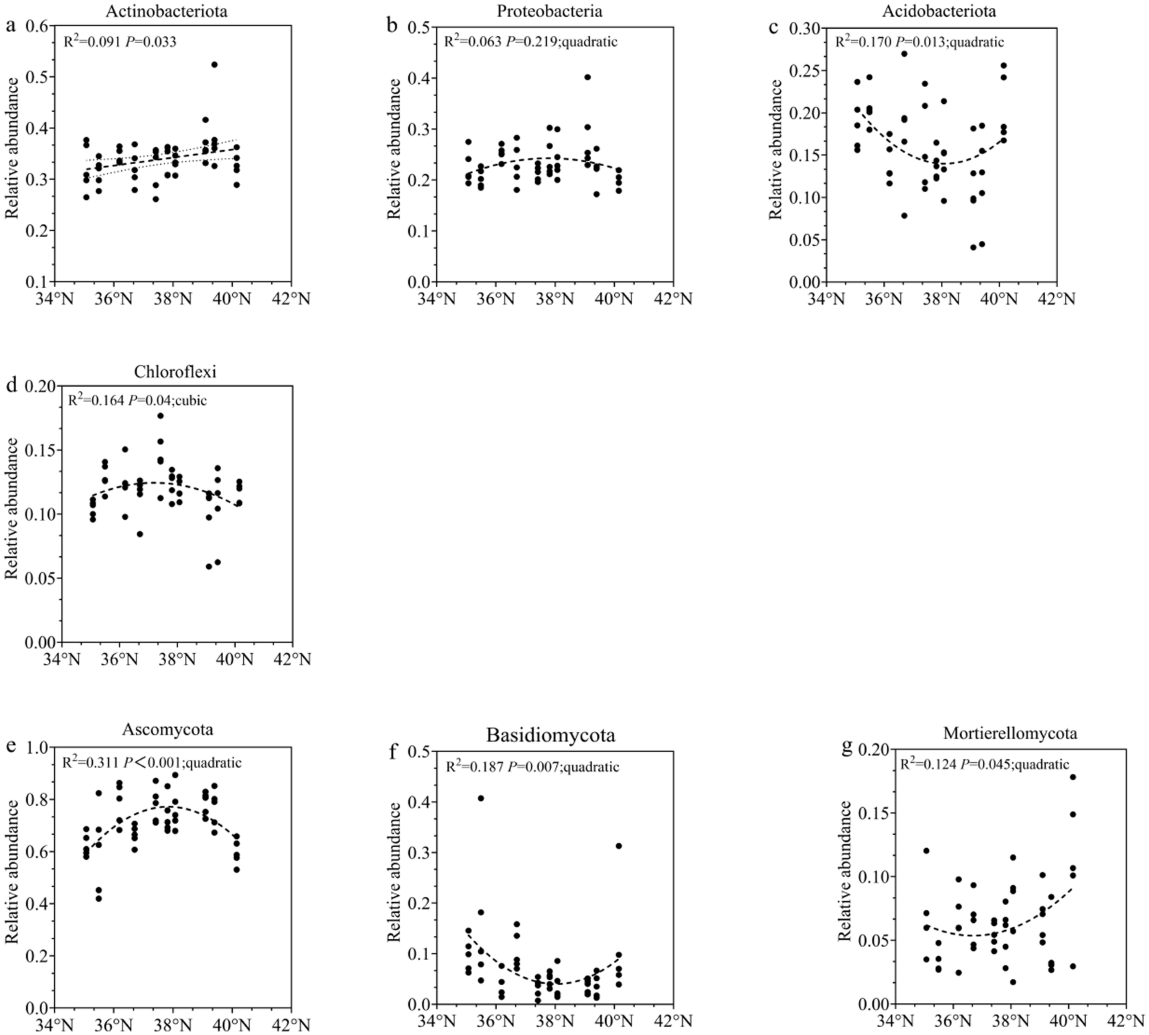
Regression analysis of soil dominant microbial communities changing with latitude gradient **(a)** *Actinobacteria*; **(b)** *Proteobacteria*; **(c)** *Acidobacteria*; **(d)** *Chloroflexita* phylum; **(e)** *Ascomycota*; **(f)** *Basidiomycota*; **(g)** *Mortierellomycota*.

The relative abundances of soil bacterial and fungal communities across 10 latitudinal sites of *Z. jujuba* habitats in the western Taihang Mountains are shown in Figures 1, 2 and Table 2. At the phylum level, the dominant bacterial taxa were Actinobacteriota (31.38–39.12%), Proteobacteria (20.35–28.64%), Acidobacteriota (10.92–20.63%), and Chloroflexi (9.97–14.59%). These four phyla collectively accounted for more than 72% of the total bacterial community. Other abundant phyla (>1%) included Gemmatimonadota (2.47–3.84%), Bacteroidota (2.07–2.91%), and Myxococcota (1.42–3.08%). Actinobacteriota varied significantly among latitudinal sites (*p* < 0.05). Its relative abundance increased gradually with increasing latitude. The northern site LQ showed significantly higher Actinobacteriota abundance than XX, YC, QX, YQ, and ZL (*p* < 0.05). Proteobacteria and Chloroflexi exhibited significant unimodal distribution patterns along the latitudinal gradient (*p* < 0.05). Proteobacteria reached its highest abundance (28.60%) at the northern site DX. This value was significantly higher than those at XX, YC, QX, TG, LQ, and ZL (*p* < 0.05). Chloroflexi reached its maximum abundance (14.60%) at the central site TG. This abundance was significantly higher than that at XX, QX, YQ, DX, LQ, and ZL (*p* < 0.05). Acidobacteriota exhibited a U-shaped distribution pattern along the latitudinal gradient. Its lowest abundance occurred at DX in the northern Taihang Mountains. This value was significantly lower than those at XX, YC, QX, and ZL (*p* < 0.05).

**Table 2:** Variance analysis of relative abundance of dominant bacteria and fungi at different latitude phyla classification levels.

Species Sites	Actinobacteriota	Proteobacteria	Acidobacteriota	Chloroflexi	Ascomycota	Basidiomycota	Mortierellomycota
XX	0.323 ± 0.02b	0.225 ± 0.01b	0.189 ± 0.01ab	0.105 ± 0bc	0.625 ± 0.02c	0.099 ± 0.02ab	0.072 ± 0.01b
YC	0.314 ± 0.01b	0.204 ± 0.01b	0.206 ± 0.01a	0.129 ± 0ab	0.601 ± 0.07c	0.164 ± 0.06a	0.033 ± 0b
GX	0.349 ± 0.01ab	0.253 ± 0.01ab	0.141 ± 0.01bcd	0.123 ± 0.01abc	0.783 ± 0.04a	0.036 ± 0.01b	0.064 ± 0.01b
QX	0.322 ± 0.02b	0.231 ± 0.02b	0.18 ± 0.03abc	0.114 ± 0.01bc	0.663 ± 0.02bc	0.106 ± 0.02ab	0.064 ± 0.01b
TG	0.321 ± 0.02b	0.214 ± 0.01b	0.164 ± 0.02abcd	0.146 ± 0.01a	0.78 ± 0.03a	0.032 ± 0.01b	0.055 ± 0b
SY	0.339 ± 0.01ab	0.245 ± 0.02ab	0.139 ± 0.01bcd	0.124 ± 0abc	0.739 ± 0.03ab	0.049 ± 0.01b	0.056 ± 0.01b
YQ	0.335 ± 0.01b	0.239 ± 0.02ab	0.15 ± 0.02abcd	0.119 ± 0bc	0.765 ± 0.04ab	0.037 ± 0.01b	0.074 ± 0.02b
DX	0.366 ± 0.01ab	0.286 ± 0.03a	0.109 ± 0.02d	0.1 ± 0.01c	0.786 ± 0.02a	0.036 ± 0.01b	0.07 ± 0.01b
LQ	0.391 ± 0.03a	0.221 ± 0.01b	0.124 ± 0.02 cd	0.109 ± 0.01bc	0.766 ± 0.03ab	0.037 ± 0.01b	0.041 ± 0.01b
ZL	0.328 ± 0.01b	0.204 ± 0.01b	0.205 ± 0.02a	0.117 ± 0bc	0.597 ± 0.02c	0.116 ± 0.05ab	0.113 ± 0.03a

Different lowercase letters indicate significant differences in dominant bacterial phyla at the *P* < 0.05 level across different latitudes. The data in the table is “mean ± standard error” (*n* = 5).

At the fungal phylum level, the dominant taxa (excluding unclassified fungi) were Ascomycota (59.67–78.56%), Basidiomycota (3.24–16.38%), and Mortierellomycota (3.32–11.29%). Together, these phyla accounted for more than 79% of the total fungal community. Other detected phyla (>0.01%) included Fungi_phy_Incertae_sedis (0.42–3.61%), Chytridiomycota (0.41–1.89%), Mucoromycota (0.13–4.24%), Glomeromycota (0.01–1.66%), Kickxellomycota (0.04–0.51%), and Blastocladiomycota (0.01–0.51%). Ascomycota exhibited a unimodal distribution, peaking at middle site TG (78.00% relative abundance). No significant differences in Ascomycota abundance were detected among GX, TG, SY, YQ, DX, and LQ (*p* > 0.05). Basidiomycota and Mortierellomycota showed U-shaped distribution patterns along the latitudinal gradient. Their lowest abundances occurred at mid-latitude sites. Basidiomycota reached its minimum abundance (3.20%) at TG in the central Taihang Mountains. Mortierellomycota showed its lowest abundance (3.30%) at YC in the southern section.

In summary, the dominant bacterial and fungal communities in the western foothills of the Taihang Mountains showed nonlinear distribution patterns along the latitudinal gradient. These patterns were primarily bimodal or trimodal.

**Figure 3:**
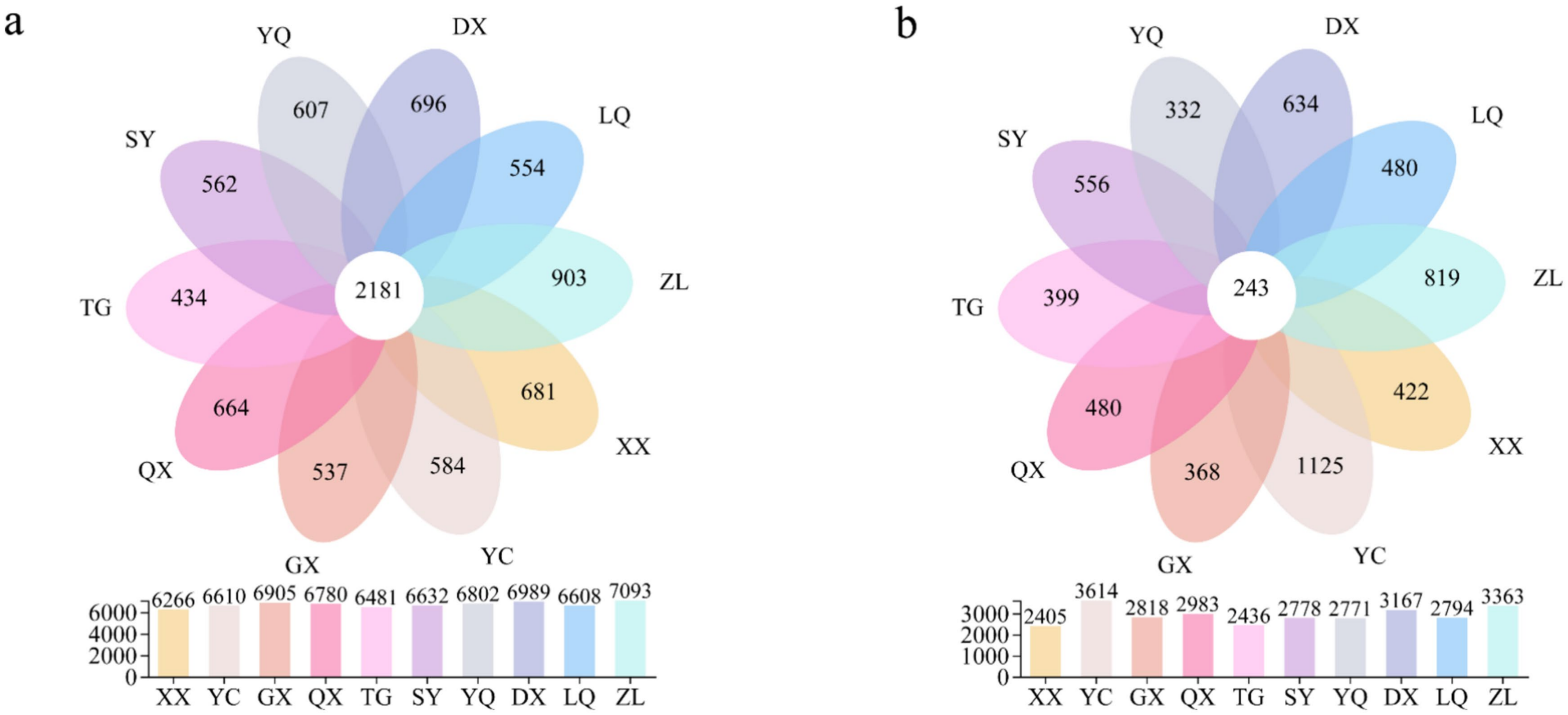
Venn diagram of soil microorganisms at different latitudes in the western foothills of Taihang Mountains: **(a)** Bacteria; **(b)** Fungi.

The distribution of soil microbial OTUs across latitudinal gradients was visualized using Venn diagrams (Figure 3). The total number of bacterial OTUs from south to north (XX to ZL) was 6,266, 6,610, 6,905, 6,780, 6,481, 6,632, 6,802, 6,989, 6,608, and 7,093. The corresponding numbers of unique OTUs were 681, 584, 537, 664, 434, 562, 607, 696, 554, and 903, respectively. A total of 2,181 bacterial OTUs were shared among all ten sites. The total numbers of fungal OTUs from south to north were 2,405, 3,614, 2,818, 2,983, 2,436, 2,778, 2,771, 3,167, 2,794, and 3,363. The numbers of unique fungal OTUs were 422, 1,125, 368, 480, 399, 556, 332, 634, 480, and 819. A total of 243 fungal OTUs were shared across all ten sites.

### 3.2 Latitudinal variation in soil microbial community alpha diversity

Statistical analysis revealed that the sequencing coverage of soil bacterial and fungal communities across 10 latitudinal gradients was greater than 0.96. This high coverage indicates that the sequencing data reliably reflected the actual composition of microbial communities in the soil samples.

**Figure 4:**
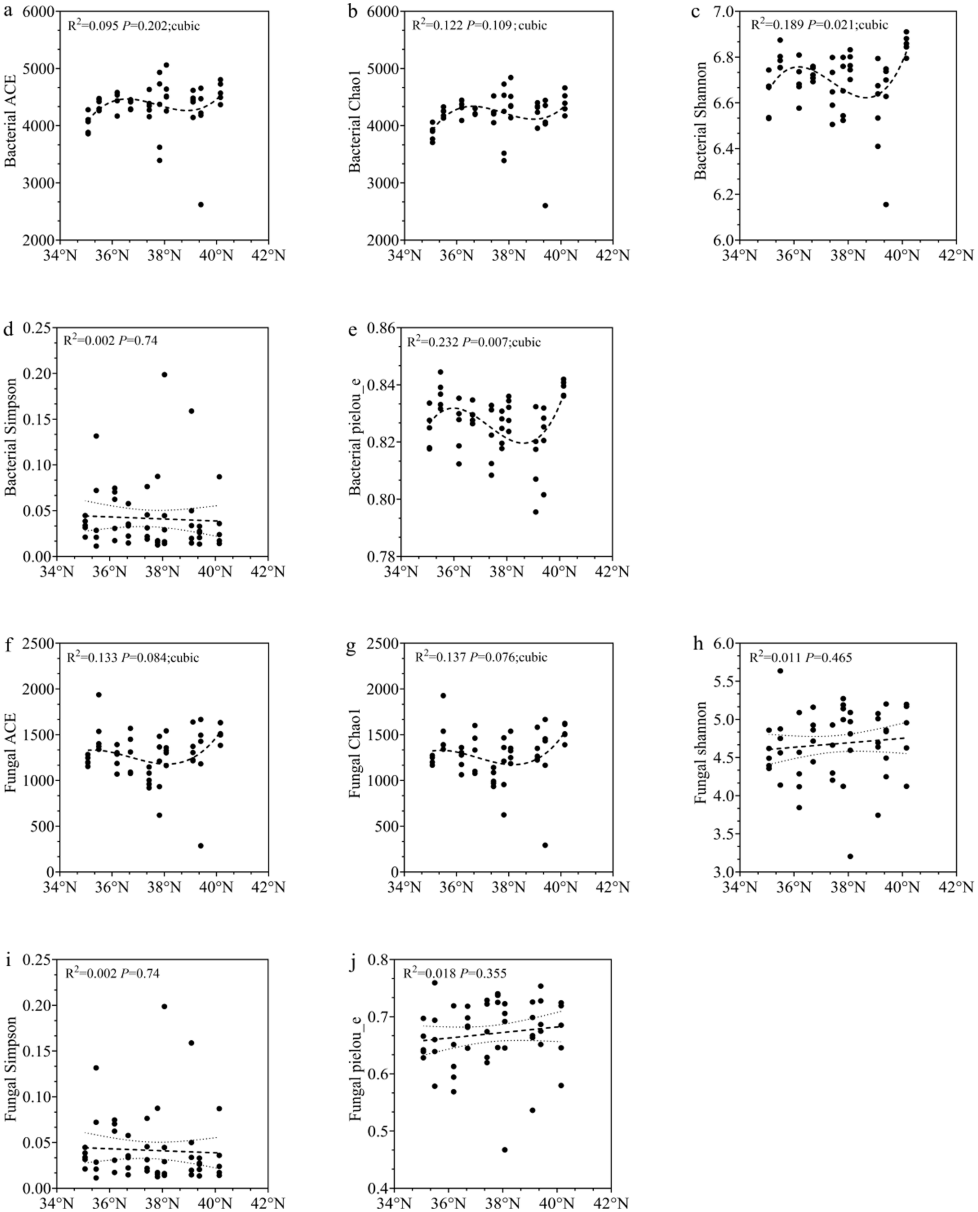
Regression analysis of soil microbial alpha diversity with latitude gradient **(a–e)** bacterial alpha diversity; (**f**–**j**) fungal alpha diversity.

The alpha diversity of soil bacterial communities was higher than that of fungal communities at all latitudes. Based on Figure 4 and Table 3, 4, several patterns were observed in the soil bacterial community. The ACE, Chao1, Shannon, and Pielou_e indices showed similar trends with increasing latitude. Their changes followed a non-linear pattern that fit a three-term equation. Specifically, the indices first increased, then decreased, and finally increased again. The ACE and Chao1 indices were highest at YQ in the middle section of the Taihang Mountains. Their values were 4595.56 and 4436.62, respectively. These values were significantly higher than those at XX and LQ (*p* < 0.05). The Shannon and Pielou_e indices reached their highest values in the northern region (ZL), at 6.86 and 0.84, respectively. These values were significantly higher than those at XX, TG, SY, DX, and LQ (*p* < 0.05). In contrast, the Simpson index showed a decreasing trend with increasing latitude. However, this change was not statistically significant (*p* > 0.05).

**Table 3:** Alpha diversity index of soil bacteria at different latitudes.

Indices Sites	ACE	Chao1	Shannon	Simpson	Pielou_e	Coverage
XX	4040.42 ± 77.53b	3874.37 ± 62.74b	6.63 ± 0.04bcd	0.0041 ± 0.0002cdef	0.82 ± 0.003bc	0.971 ± 0.0005a
YC	4383.17 ± 43.39ab	4229.67 ± 34.33ab	6.79 ± 0.02ab	0.0032 ± 0.0001ef	0.84 ± 0.002a	0.969 ± 0.0004a
GX	4460.13 ± 77.28ab	4333.94 ± 65.16ab	6.69 ± 0.04abcd	0.0043 ± 0.0004cde	0.82 ± 0.004bc	0.967 ± 0.0006a
QX	4378.75 ± 38.18ab	4243.5 ± 24.7ab	6.73 ± 0.01abcd	0.0038 ± 0.0001def	0.83 ± 0.001ab	0.968 ± 0.0005a
TG	4360.92 ± 78.74ab	4249.1 ± 75.61ab	6.66 ± 0.05bcd	0.005 ± 0.0005abc	0.82 ± 0.005bc	0.968 ± 0.0006a
SY	4210.68 ± 302.73ab	4083.58 ± 268.78ab	6.66 ± 0.06bcd	0.0054 ± 0.0004ab	0.82 ± 0.002bc	0.97 ± 0.003a
YQ	4595.56 ± 131.91a	4436.62 ± 117.4a	6.77 ± 0.02abc	0.0038 ± 0.0002def	0.83 ± 0.002ab	0.966 ± 0.0013a
DX	4422.37 ± 77.51ab	4251.33 ± 78.51ab	6.61 ± 0.07 cd	0.0058 ± 0.0005a	0.81 ± 0.006c	0.968 ± 0.0005a
LQ	4031.21 ± 362.78b	3899.12 ± 333.69b	6.59 ± 0.11d	0.0046 ± 0.0005bcd	0.82 ± 0.005bc	0.971 ± 0.0032a
ZL	4592.99 ± 78.47a	4409.02 ± 85.21a	6.86 ± 0.02a	0.003 ± 0.0001f	0.84 ± 0.001a	0.967 ± 0.0008a
F	1.51	1.748	3.083	7.245	4.538	1.398
p	0.184	0.117	0.008	0	0.001	0.227

Different lowercase letters on the same line indicate significant differences in each indicator at the *P* < 0.05 level. The data in the table is “mean ± standard error” (*n* = 5).

**Table 4:** Alpha diversity index of soil fungi at different latitudes.

Indices Sites	ACE	Chao1	Shannon	Simpson	Pielou_e	Coverage
XX	1215.19 ± 22.5ab	1211.25 ± 21.57ab	4.54 ± 0.09a	0.0339 ± 0.0039a	0.65 ± 0.012a	0.995 ± 0.0001abc
YC	1517.28 ± 110.13a	1516.85 ± 107.64a	4.79 ± 0.25a	0.0529 ± 0.0223a	0.67 ± 0.03a	0.994 ± 0.0006bc
GX	1247.51 ± 55.23b	1236.3 ± 52.83ab	4.38 ± 0.21a	0.0511 ± 0.0114a	0.63 ± 0.026a	0.995 ± 0.0004abc
QX	1299.58 ± 97.16ab	1314.53 ± 101.58ab	4.82 ± 0.12a	0.0327 ± 0.0073a	0.69 ± 0.012a	0.995 ± 0.0007abc
TG	1021.88 ± 41.14b	1024.73 ± 38.72b	4.6 ± 0.15a	0.0387 ± 0.0105a	0.67 ± 0.023a	0.997 ± 0.0004a
SY	1122.6 ± 155.56b	1123.18 ± 151.61b	4.94 ± 0.21a	0.0299 ± 0.0144a	0.72 ± 0.018a	0.996 ± 0.0008ab
YQ	1338.38 ± 60.91ab	1329.39 ± 59.92ab	4.53 ± 0.34a	0.0605 ± 0.035a	0.65 ± 0.047a	0.994 ± 0.0006bc
DX	1351.23 ± 77.25ab	1331.49 ± 66.38ab	4.63 ± 0.24a	0.0554 ± 0.0266a	0.66 ± 0.033a	0.994 ± 0.0006bc
LQ	1210.97 ± 243.96ab	1202.54 ± 241.09ab	4.73 ± 0.16a	0.0241 ± 0.0033a	0.7 ± 0.018a	0.995 ± 0.0013abc
ZL	1530.68 ± 46.68a	1527.53 ± 42.73a	4.82 ± 0.2a	0.0356 ± 0.0134a	0.67 ± 0.027a	0.994 ± 0.0005c
F	2.277	2.351	0.68	0.544	0.951	1.946
*P*	0.041	0.036	0.706	0.816	0.487	0.079

Different lowercase letters on the same line indicate significant differences in each indicator at the *P* < 0.05 level. The data in the table is “mean ± standard error” (*n* = 5).

In the soil fungal communities, the ACE and Chao1 indices showed similar trends. They followed a U-shaped pattern along the latitudinal gradient. Specifically, the indices first decreased and then increased. Both indices reached their lowest values at TG in the middle section. These values were significantly lower than those at YC and ZL (*p* < 0.05). In contrast, the Shannon and Pielou_e indices gradually increased with latitude. The Simpson index showed a decreasing trend. However, none of these three indices differed significantly among latitudes (*p* > 0.05). Overall, no consistent latitudinal pattern was observed in the alpha diversity of soil bacterial and fungal communities. The overall variation appeared random. Microbial richness indices were generally higher in the middle regions. In contrast, diversity and evenness indices often reached their highest values in the northern regions.

### 3.3 Latitudinal variation in soil microbial community structure

**Figure 5:**
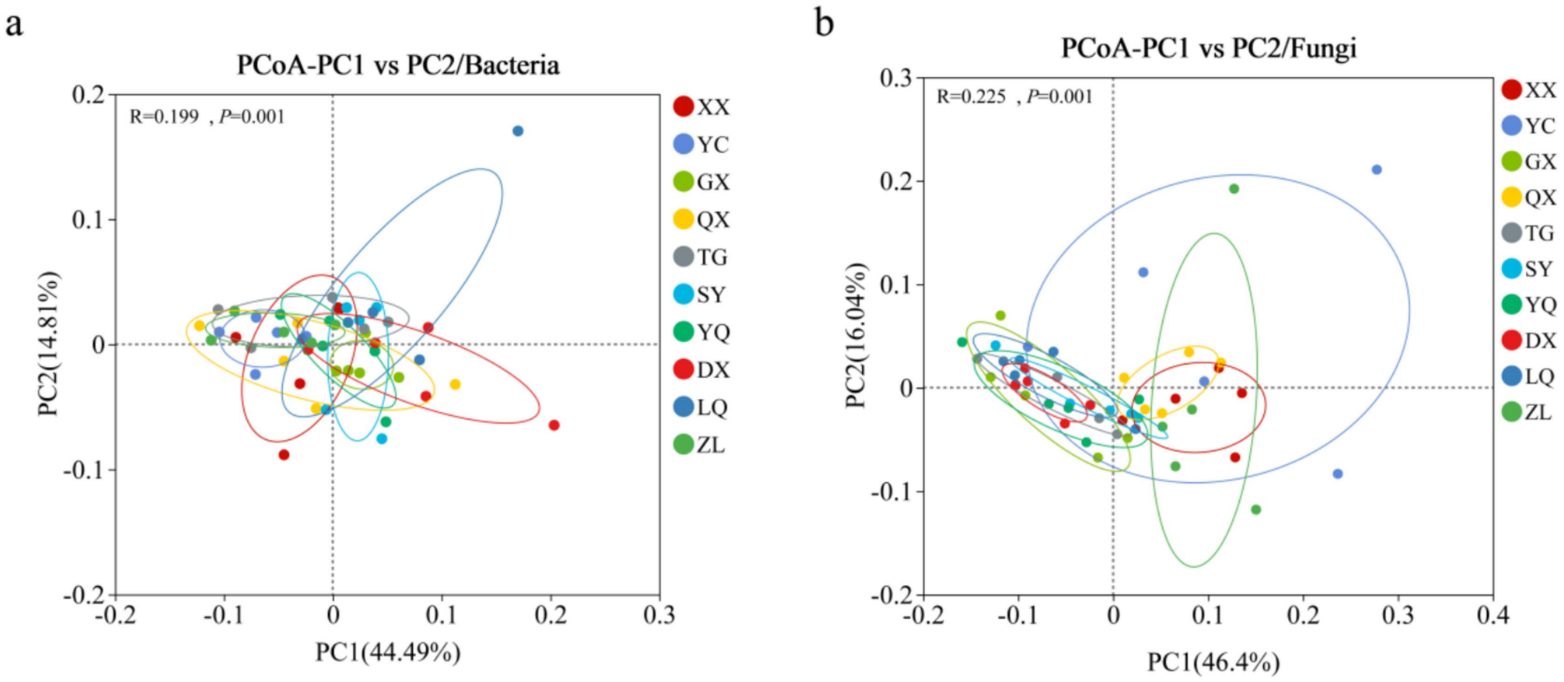
PCoA results of soil microorganisms in *Z. jujuba* shrubs at different latitudes in Taihang Mountains: **(a)** Bacterial PCoA; **(b)** fungal PCoA.

**Figure 6:**
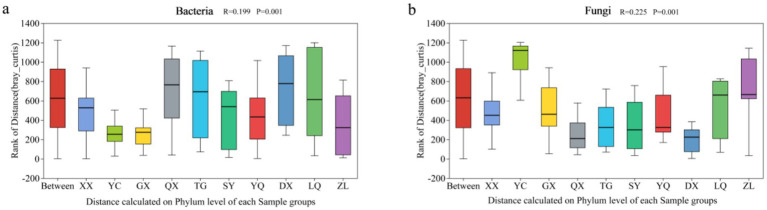
Beta diversity difference analysis of soil microbial communities in *Z. jujuba* shrublands at different latitudes in Taihang Mountains: **(a)** Bacteria; **(b)** Fungi.

Based on the PCoA analysis results (Figure 5), we found that the latitudinal gradient has a highly significant influence on the composition and structure of both soil bacterial and fungal communities (*p* = 0.001). Among them, the latitudinal gradient explains a higher proportion of variation in fungal community differences (*R* = 0.225) than in bacterial community differences (*R* = 0.199). This indicates that, compared to bacteria, fungal community structure exhibits a more distinct spatial differentiation pattern along the latitudinal gradient. In other words, as latitude increases from low to high, the composition of fungal communities changes more dramatically and uniquely than that of bacterial communities. Based on the analysis of beta diversity intergroup differences (Figure 6), bacterial communities show statistical significance (*R* = 0.19, *p* = 0.001), indicating a significant latitudinal influence. However, the median values of the box plots reveal a more complex distribution pattern in the degree of structural differences across latitudes. There is no clear or consistent monotonic increasing trend. These results suggest that latitude is not the sole or dominant factor driving spatial variation in bacterial communities. Soil factors may play a more important role in shaping bacterial community differences. In contrast, the median values of the box plots for fungal communities at different latitudes show a clear and gradually increasing trend with rising latitude. This trend is strongly supported by statistical analysis (*R* = 0.225, *p* = 0.001). This directly demonstrates a significant latitudinal pattern in fungal community beta diversity. Fungal communities become increasingly distinct from those in lower-latitude regions as latitude increases. In summary, the composition and spatial variation of fungal communities are far more sensitive and regular in response to the latitudinal gradient compared to bacterial communities.

The results of permutation multivariate analysis of variance (PERMANOVA) showed that grouping according to different latitude factors was the primary factor influencing soil bacterial and fungal community beta diversity (Table 5). The explanatory rates were 42.90% for bacterial communities and 37.96% for fungal communities. In addition, Lat, Al, MAP, pH, EC, CLA, TC, TN, SOC, NH_4_^+^-N, C/N, LWC, and LC were the main factors affecting soil bacterial beta diversity. MAP, CLA, SIL, SAN, TC, TN, and SOC were the main factors affecting soil fungal beta diversity.

**Table 5:** PERMANOV on the effects of different environmental factors on soil bacterial and fungal communities at the phylum level.

Indices	Bacteria			Fungi		
	*F*	*R* ^2^	*P*	*F*	*R* ^2^	*P*
Group by grouping factors	3.338	0.429	**0.001**	2.719	0.380	**0.001**
Long	0.957	0.020	0.385	0.956	0.020	0.381
Lat	3.486	0.068	**0.019**	2.651	0.052	0.057
Al	2.924	0.057	**0.046**	2.198	0.044	0.100
MAT	1.630	0.033	0.165	1.242	0.025	0.270
MAP	10.308	0.177	**0.001**	3.834	0.074	**0.016**
BD	0.596	0.012	0.622	0.322	0.007	0.867
SWC	0.578	0.012	0.499	0.724	0.015	0.443
pH	5.154	0.097	**0.006**	1.714	0.034	0.162
EC	3.701	0.072	**0.017**	2.689	0.053	0.051
CLA	3.460	0.067	**0.027**	4.214	0.081	**0.011**
SIL	2.206	0.044	0.104	4.637	0.088	**0.009**
SAN	2.665	0.053	0.070	5.202	0.098	**0.006**
TC	5.470	0.102	**0.004**	6.166	0.114	**0.002**
TN	9.766	0.169	**0.001**	6.562	0.120	**0.001**
TP	0.452	0.009	0.654	2.056	0.041	0.105
SOC	5.110	0.096	**0.009**	3.159	0.062	**0.036**
NH_4_^+^-N	1.226	0.025	0.295	1.430	0.029	0.224
NO_3_^—^-N	0.096	0.002	0.977	0.557	0.011	0.616
C/N	3.816	0.074	**0.013**	1.420	0.029	0.208
C/P	2.067	0.041	0.096	1.849	0.037	0.139
N/P	1.041	0.021	0.375	1.157	0.024	0.315
LWC	4.876	0.092	**0.008**	1.580	0.032	0.198
LC	0.741	0.015	0.520	0.102	0.002	0.979
LN	0.307	0.006	0.844	0.961	0.020	0.372
LiWC	2.618	0.052	0.083	0.941	0.019	0.340
LiC	2.659	0.052	0.058	2.328	0.046	0.058
LiN	0.275	0.006	0.876	0.931	0.019	0.408

Bold values indicate differences at the *P* < 0.05 level remarkable.

### 3.4 Latitudinal variation in soil microbial community assembly processes

**Figure 7:**
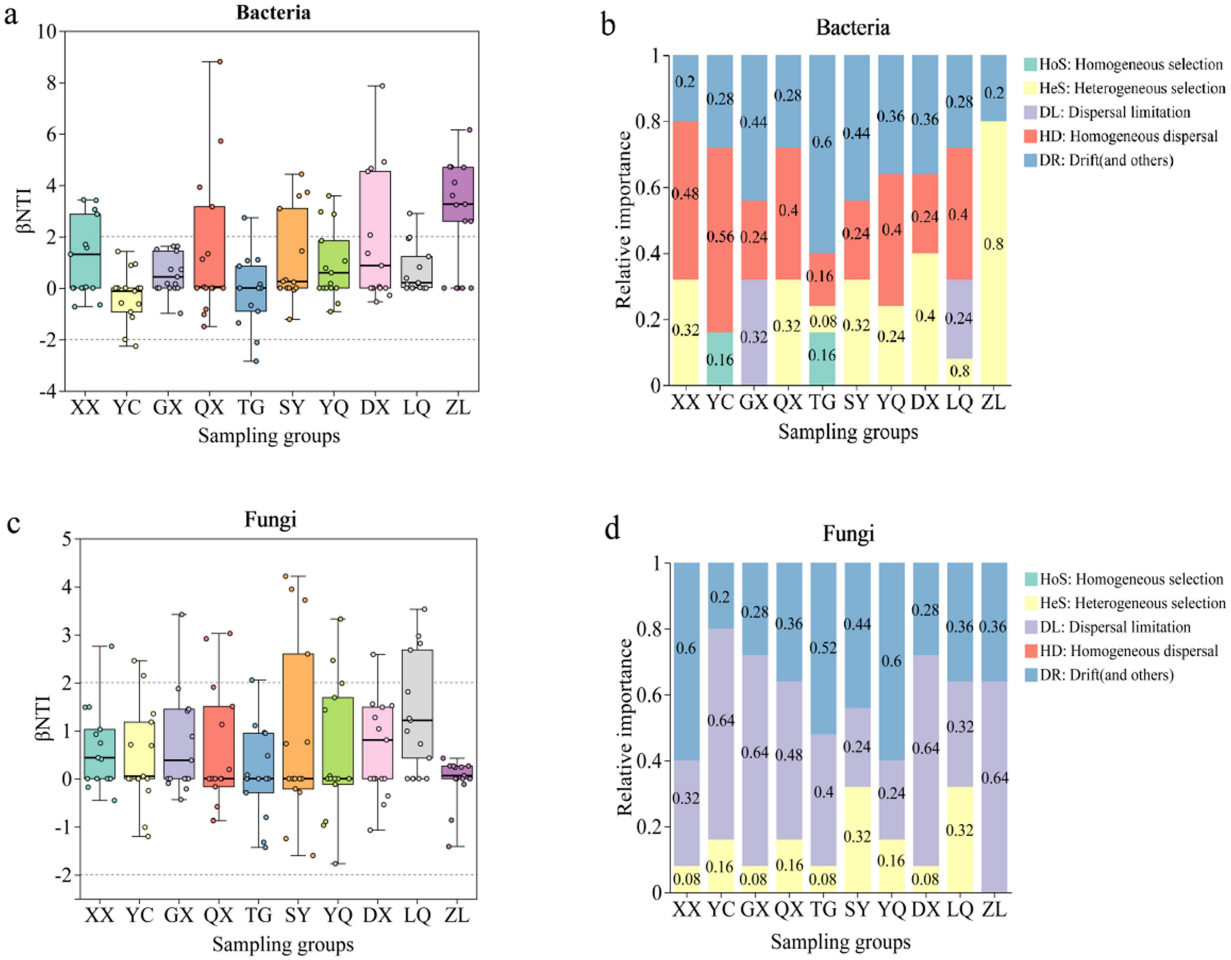
Null model analysis of soil microbial communities along a latitudinal gradient on the western foothills of the Taihang Mountains. **(a)** Bacterial βNTI; **(b)** Relative importance of bacteria; **(c)** Fungal βNTI; **(d)** Relative importance of Fungi.

As shown in Figure 7a, in the *Z. jujuba* shrublands sampling sites on the western foothills of the Taihang Mountains, half of the βNTI values were above 2, while the other half were below 2. Combined with the results of the null model (Figure 7b), bacterial community assembly in most sampling sites was dominated by stochastic processes. The proportion of dominance by stochastic processes fluctuated with increasing latitude. In the southern region, GX was entirely influenced by stochastic processes, accounting for 100%. Drift and other ecological processes (DR: Drift and others) served as the primary driver, accounting for 44%. In the northern region, only ZL was determined by heterogeneous selection (HeS) among deterministic processes. The proportion reached as high as 80%. Figure 7c showed that only two sampling sites had βNTI values above 2. The null model indicated that fungal community assembly was primarily dominated by stochastic processes, with proportions exceeding 68% in all cases. In ZL in the northern region, stochastic processes completely controlled fungal assembly. Diffusion limitation accounted for over 64%. The other nine sampling sites along the latitude gradient were also influenced by deterministic processes to a small extent. Among them, SY and LQ were more significantly affected by heterogeneous selection (HeS), both accounting for 32% (Figure 7d). Environmental factors exhibit weak selective effects on microbial composition across different sampling sites on the western foothills. Spatial diffusion and stochastic events dominate community assembly. This indicates that regional environmental heterogeneity imposes relatively limited constraints on the distribution of bacteria and fungi.

**Figure 8:**
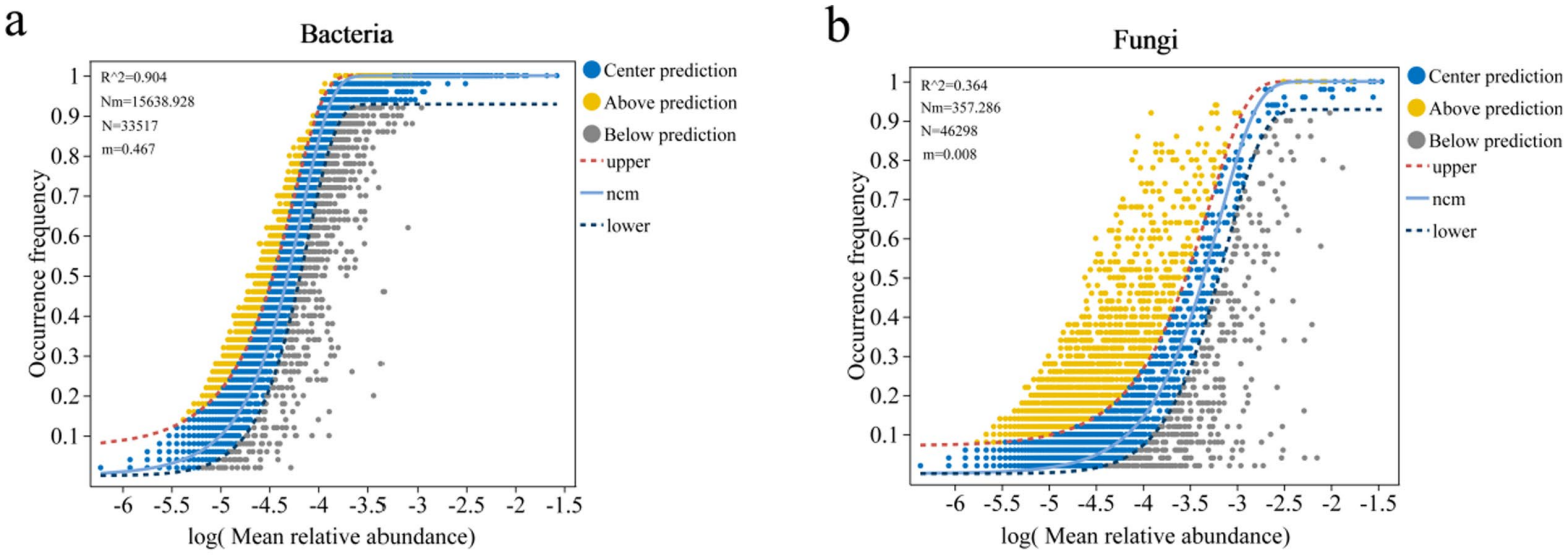
Analysis of soil microbial community assembly process using neutral community model (NCM): **(a)** Bacteria and **(b)** Fungal. R^2^ represents the overall goodness of fit of the neutral community model. A higher R^2^ indicates a closer fit to the neutral model, indicating that the construction of the community is more affected by stochastic processes and less affected by deterministic processes. *N* describes the meta community size, which is the total abundance of all OTUs in each sample. The value of *m* quantifies the migration rate at the community level, which is uniform for each community member (regardless of species). The smaller the value of *m*, the more restricted the diffusion of species in the entire community. Conversely, the higher the value of *m*, the lower the diffusion restriction of species. *Nm* is the product of the size of the metacommunity (*N*) and the migration rate (*m*) (*Nm* = N*m), quantifying the estimation of diffusion between communities and determining the correlation between frequency of occurrence and relative abundance of regions.

The neutral community model (NCM) assumes that the assembly of microbial communities is mainly influenced by random diffusion and neutral processes rather than specific environmental factors. It is therefore widely used to analyze the assembly mechanisms of microbial communities. In this study, the NCM was applied to comprehensively analyze OTU-level data of microbial communities across different latitudinal gradients. It was also used to conduct an in-depth analysis of bacterial and fungal communities at different latitudes (Figure 7). The overall goodness of fit (*R*^2^) and migration rate (m) of the neutral model were compared between bacteria and fungi. The results showed that the goodness of fit for bacteria (*R*^2^ = 0.904) was significantly higher than that for fungi (*R*^2^ = 0.364). This indicates that bacterial community assembly is more influenced by stochastic processes and less by deterministic processes, while fungal communities show the opposite pattern. The bacterial migration rate (*m* = 0.467) was much higher than that of fungi (*m* = 0.008). This indicates that bacterial communities are less restricted by diffusion, whereas fungal communities are strongly limited by diffusion.

### 3.5 Latitudinal variation in soil physicochemical properties and plant factors

The soil physicochemical properties of *Z. jujuba* shrublands on the western foothills of the Taihang Mountains exhibited distinct distribution patterns along the latitudinal gradient (Table 6). Soil bulk density, soil water content, soil electrical conductivity, soil nitrate nitrogen, and soil carbon-to-nitrogen ratio displayed a unimodal trend with increasing latitude. These properties peaked in the middle and northern sections of the Taihang Mountains, primarily in the TG, SY, and DX regions. The clay content, total carbon, total nitrogen, soil organic carbon, soil carbon-to-phosphorus ratio, and nitrogen-to-phosphorus ratio exhibited a U-shaped distribution pattern with latitude. Their minimum values occurred in the mid-section of the Taihang Mountains, mainly in the TG and SY regions. Soil silt content and soil ammonium nitrogen content showed a decreasing trend with increasing latitude. Both indicators reached their highest values at XX in the southern Taihang Mountains. Soil sand content and soil total phosphorus content increased along the latitudinal gradient. Soil sand content was highest in DX in the northern section, significantly higher than the other nine sites (*p* < 0.05). The highest soil total phosphorus content was observed at ZL in the northern Taihang Mountains. This value was 33.33% higher than the lowest value at YC. Soil pH along the latitudinal gradient showed a non-linear trend fitted by a three-term equation. GX in the southern section had a significantly higher pH than YC, QX, SY, YQ, DX, and ZL (*p* < 0.05).

**Table 6:** Changes in soil physical and chemical properties at different latitudes.

Samples Indices	Southern section of Taihang Mountains			Middle section of Taihang Mountains				North section of Taihang Mountains		
	XX	YC	GX	QX	TG	SY	YQ	DX	LQ	ZL
BD/(g·cm^−3^)	0.97 ± 0.03b	1.19 ± 0.05a	1.1 ± 0.08ab	0.95 ± 0.04b	1.09 ± 0.02ab	1.12 ± 0.04ab	1.01 ± 0.06b	1.2 ± 0.06a	0.96 ± 0.07b	0.96 ± 0.05b
SWC/(%)	13.88 ± 1.85ab	14.48 ± 0.82ab	17.57 ± 1ab	8.23 ± 1.03b	26.59 ± 14.61a	17 ± 1.14ab	9.43 ± 0.74b	18.35 ± 2.26ab	9.88 ± 0.58b	8.68 ± 1.16b
pH	8.02 ± 0.1a	7.1 ± 0.03d	8.13 ± 0.03a	7.42 ± 0.08c	8.03 ± 0.04a	7.73 ± 0.05b	7.72 ± 0.04b	7.71 ± 0.08b	7.91 ± 0.15ab	7.75 ± 0.08b
EC/(mS·cm^−1^)	0.17 ± 0.01b	0.07 ± 0.01d	0.15 ± 0.01bc	0.1 ± 0.02 cd	0.14 ± 0bc	0.26 ± 0.03a	0.19 ± 0.01b	0.16 ± 0.02b	0.18 ± 0.02b	0.15 ± 0.01bc
CLA/(%)	7.7 ± 0.31ab	9.06 ± 0.78a	6.12 ± 0.55bcd	5.26 ± 0.59 cd	7.79 ± 0.4ab	5.23 ± 0.29 cd	8 ± 1.22ab	4.62 ± 0.87d	4.42 ± 0.13d	7.42 ± 1.2abc
SIL/(%)	82.96 ± 0.96a	77.32 ± 4.03ab	78.79 ± 3.66ab	79.72 ± 3.17a	78.96 ± 1.84ab	79.2 ± 1.49ab	80.11 ± 3.15a	53.37 ± 6.45c	68.51 ± 1.69b	75.57 ± 3.18ab
SAN/(%)	9.34 ± 1.24c	13.29 ± 4.51c	15.09 ± 4.18bc	15.02 ± 3.7bc	13.25 ± 2.22c	15.57 ± 1.73bc	11.9 ± 4.35c	41.87 ± 7.22a	27.07 ± 1.81b	17.01 ± 3.99bc
TC/(g·kg^−1^)	14.15 ± 0.76 cd	19.56 ± 0.88b	8.05 ± 0.48f	13.01 ± 0.65de	15.99 ± 0.85c	4.33 ± 0.31 g	14.31 ± 0.78 cd	11.01 ± 0.63e	14.9 ± 1.05 cd	26.17 ± 1.1a
TN/(g·kg^−1^)	0.75 ± 0.02bc	0.81 ± 0.03b	0.49 ± 0.02f	0.64 ± 0.02de	0.28 ± 0.01 g	0.29 ± 0.02 g	0.59 ± 0.03e	0.35 ± 0.02 g	0.69 ± 0.04 cd	0.91 ± 0.03a
TP/(g·kg^−1^)	0.25 ± 0.04ab	0.21 ± 0.02b	0.23 ± 0.02ab	0.24 ± 0.01ab	0.28 ± 0.03ab	0.26 ± 0.05ab	0.27 ± 0.02ab	0.23 ± 0.12ab	0.28 ± 0.02ab	0.28 ± 0.02a
SOC/(g·kg^−1^)	14.53 ± 1.36bcd	28.31 ± 3.64a	10.61 ± 0.27 cd	11.07 ± 0.64 cd	16.89 ± 1.76bc	8.71 ± 0.75d	14.47 ± 0.97bcd	16.74 ± 2.23bc	20.68 ± 2.63b	34.35 ± 3.66a
NH_4_^+^-N/(mg·kg^−1^)	45.57 ± 3.69a	15.71 ± 1.62c	16.9 ± 1.4c	16.87 ± 1.49c	14.94 ± 1.1c	13.33 ± 1.25c	31.39 ± 3.44b	14.66 ± 1.21c	18.99 ± 1.14c	15 ± 1.13c
NO_3_^—^-N/(mg·kg^−1^)	2.02 ± 0.27c	2.13 ± 0.29c	1.92 ± 0.3c	3.46 ± 0.53abc	2.48 ± 0.33bc	4.17 ± 0.87a	4.1 ± 0.64ab	2.61 ± 0.27abc	2.72 ± 0.67abc	2.86 ± 0.41abc
C/N	18.81 ± 0.68def	24.36 ± 1.84 cd	16.47 ± 1.48ef	20.24 ± 0.82def	57.26 ± 1.98a	15.35 ± 1.92f	24.35 ± 1.87 cd	32.32 ± 2.54b	21.89 ± 1.89de	29.03 ± 1.74bc
C/P	59.45 ± 7.4ab	93.36 ± 8.07a	35.59 ± 1.87b	53.72 ± 2.79ab	58.28 ± 2.99ab	17.24 ± 1.56b	52.51 ± 3.89ab	86.04 ± 45.53a	53.35 ± 3.7ab	93.25 ± 5.94a
N/P	3.13 ± 0.29a	3.84 ± 0.21a	2.2 ± 0.15abc	2.66 ± 0.12ab	1.02 ± 0.03c	1.18 ± 0.16bc	2.17 ± 0.12abc	2.79 ± 1.53ab	2.46 ± 0.13abc	3.21 ± 0.06a

Different lowercase letters on the same line indicate significant differences in various indicators at the *P* < 0.05 level. The data in the table is “mean ± standard error” (*n* = 5).

**Figure 9:**
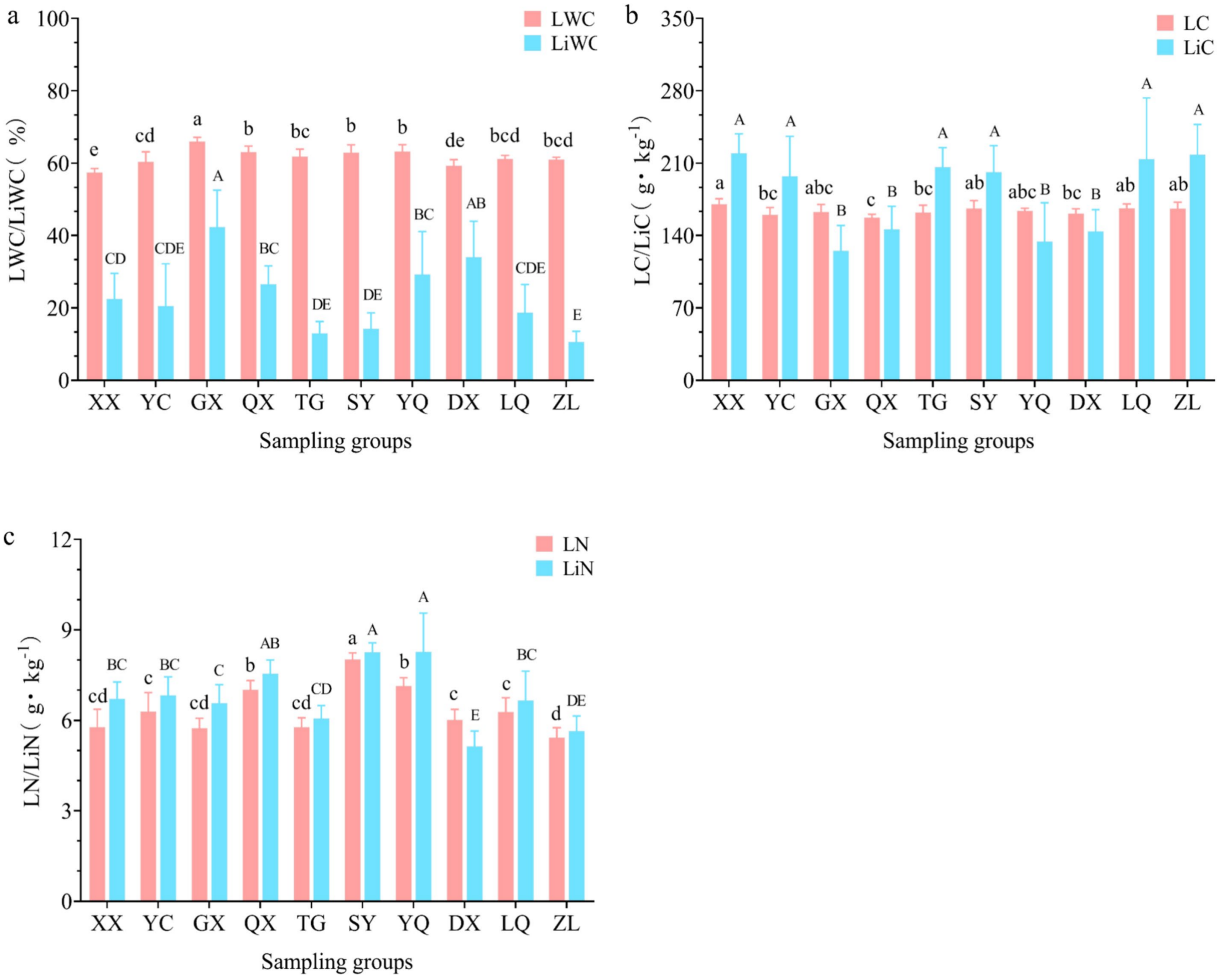
Distribution characteristics of plant leaves and litter at different latitudes: **(a)** Water content; **(b)** carbon content; **(c)** nitrogen content.

The leaf water content (LWC), litter water content (LiWC), leaf nitrogen content (LN), and litter nitrogen content (LiN) showed significant variations across latitudinal gradients (*p* < 0.05). These variables exhibited a unimodal distribution pattern with increasing latitude. Specifically, LWC and LiWC peaked in GX in the southern section of the Taihang Mountains, measuring 66.10 and 42.43%, respectively. These values were significantly higher than those at other latitudes (*p* < 0.05) (Figure 9a). Meanwhile, LN and LiN peaked in SY in the mid-section of the Taihang Mountains. Their maximum values were 8.03 g·kg^−1^ and 8.28 g·kg^−1^, respectively (Figure 9c). In contrast, leaf carbon content (LC) and litter carbon content (LiC) followed a U-shaped distribution pattern along the latitudinal gradient. They initially decreased and then increased with rising latitude. The carbon content of plant leaves (LC) reached its lowest value (170.71 g·kg^−1^) in the middle section of the Taihang Mountains. The carbon content of litter (LiC) was lowest (219.97 g·kg^−1^) in the southern section (Figure 9b).

### 3.6 Relationships between soil microbial communities and environmental factors across different latitudinal gradients

Spearman correlation analysis revealed significant correlations between the abundance of soil microbial communities and four categories of factors: spatial, climatic, soil nutrient, and vegetation-related factors (*p* < 0.05; Supplementary Appendix Figure 2). Among the top three dominant bacterial phyla, Actinobacteriota showed a significant positive correlation with soil EC and a negative correlation with TC (*p* < 0.05). Proteobacteria was positively correlated with SWC, LWC, and LiWC, but negatively correlated with soil TC, TN, TP, SOC, and the C/P ratio (*p* < 0.05). Acidobacteriota exhibited positive correlations with MAP, soil TC, and TN (*p* < 0.05).

Regarding the dominant fungal phyla, Ascomycota correlated positively with Long, Lat, and soil EC, but negatively with MAP (*p* < 0.05). Basidiomycota was positively correlated with MAP but negatively correlated with Lat, soil EC, TP, and plant LN (*p* < 0.05). Mortierellomycota showed positive correlations with SWC, the N/P ratio, and LC (*p* < 0.05). Redundancy analysis (RDA) was performed to assess the influence of major environmental and vegetation factors on soil microbial community composition in *Z. jujuba* shrublands across latitudinal gradients in the Taihang Mountains (Supplementary Appendix Figure 2).

**Figure 10:**
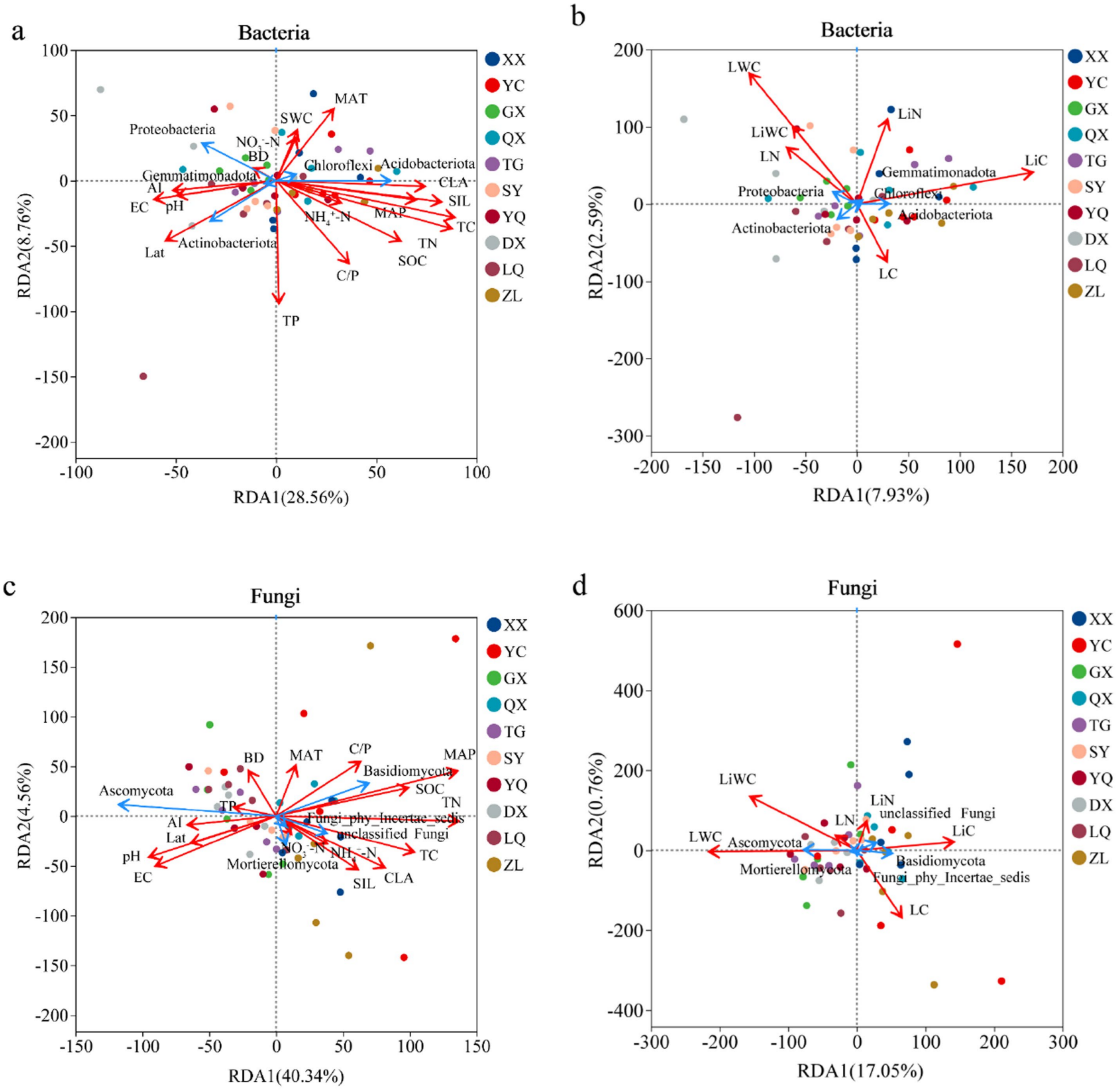
Redundancy analysis of soil microbial community composition by main environmental and vegetation factors at different latitudes in Taihang Mountains. **(a,b)** RDA analysis of bacterial communities; **(c,d)** RDA analysis of fungal communities. BD, soil bulk density; SWC, soil moisture content; pH, soil acidity and alkalinity; EC, soil electrical conductivity; CLA, soil clay particles; SIL, soil silt particles; TC, soil total carbon; TN, soil total nitrogen; TP, soil total phosphorus; SOC, soil organic carbon; NH_4_^+^-N: Soil ammonium nitrogen; NO_3_-N, soil nitrate nitrogen; C/P, soil carbon to phosphorus ratio; LWC, leaf moisture content; LC, leaf carbon content; LN, leaf nitrogen content; LiWC, litter water content; LiC, litter carbon content; LiN, litter nitrogen content.

In the RDA of soil bacterial community composition, the first two axes explained 37.32 and 10.52% of the total variation, respectively (Figures 10a,b). Soil physicochemical factors that significantly impacted bacterial communities included TN, SAN, TP, and TC, with contribution rates of 29.50, 18.10, 12.60, and 10.40%, respectively. LiWC and LWC were the main plant factors driving soil bacterial communities, with contribution rates of 45.00 and 22.60%, respectively. In the RDA of soil fungal community composition, the first two axes explained 44.90 and 17.81% of the total variation, respectively (Figures 10c,d). Soil physicochemical factors significantly affecting fungal communities included MAP, TN, NO_3_^—^-N, and SIL, with contribution rates of 42.50, 11.30, 5.60, and 4.90%, respectively. LWC and LiN were the main vegetation factors driving changes in soil fungal communities, with contribution rates of 63.20 and 16.80%, respectively.

## 4 Discussion

### 4.1 Response of soil microbial community composition to latitudinal variation

Soil microorganisms can sensitively respond to subtle changes in environmental factors. They adjust their community composition and structure accordingly (Ma, 2024). From the Venn plots of sampling points at different latitudes (Figure 3), the total number of bacterial OTUs and their unique OTUs were significantly higher than those of fungi. This finding aligns with studies in other habitats such as desert grasslands, wetlands, plateaus, and forests (Yu et al., 2020; Zhang et al., 2018). This demonstrates that soil bacteria, as dominant species, exhibit stronger reproductive capabilities and greater environmental adaptability compared to fungi.

Studies have demonstrated that variations in latitude lead to distinct changes in soil physicochemical properties and climatic factors. These changes consequently alter the composition and activity of soil microbial communities (Noah and Robert, 2006; Lauber et al., 2009; Blau et al., 2018; Obayomi et al., 2021; Zhang et al., 2018). This study found that the dominant microbial groups at the phylum level in *Z. jujuba* shrublands soils on the western foothills of the Taihang Mountains were consistent with the results reported by Ma (2024), Zhan et al. (2018), and Zhang et al. (2022). Differences were mainly reflected in variations in microbial relative abundance. Specifically, the relative abundance of Actinobacteria increased with latitude. Many taxa within this phylum can survive in low-temperature environments and tolerate arid conditions. This allows them to adapt to the colder climate in the northern Taihang Mountains (Zhang et al., 2017). In contrast, Proteobacteria and Chloroflexi reached peak abundance in the central-southern regions of the Taihang Mountains as latitude increased. This pattern may be attributed to the higher soil C/N ratio in these areas. This pattern may be attributed to the higher soil C/N ratio in these areas. A carbon-rich environment promotes the growth of these bacterial groups (Yang et al., 2022). Additionally, the mid- and southern sections of the study area had higher soil moisture content, poor aeration, and lower oxygen levels. These conditions created more favorable environments for Chloroflexi. Acidobacteria, known as slow-growing oligotrophs that thrive in acidic environments, typically decrease in abundance with increasing nitrogen availability (Fierer et al., 2007). They usually increase with declining soil pH (Jones et al., 2009). However, in this study, the variation in Acidobacteria abundance aligned with trends in soil total nitrogen (TN). It showed no significant correlation with pH. The reasons for this difference may include the following. First, certain Acidobacteria species can fully utilize nitrogen in the soil to maintain their growth and reproduction. Higher nitrogen content favors the growth and development of this population (Tong et al., 2024). Second, in this study, average annual precipitation was significantly positively correlated with Acidobacteria. High precipitation is usually accompanied by higher plant productivity. This can increase litter input and soil organic carbon (Zhu et al., 2024; Chen et al., 2016). Some groups of Acidobacteria are good at degrading complex organic matter and can benefit from rainy environments (Kielak et al., 2016).

Among the dominant fungal phyla, the relative abundance of Ascomycota was higher in the central Taihang Mountains but lower in the southern and northern regions. This spatial pattern may be attributed to optimal hydrothermal conditions in the central zone, which favor the growth and reproduction of most Ascomycota taxa. Additionally, the well-developed root systems of *Z. jujuba* shrublands can access deep soil water, while the shrub canopy reduces surface evaporation. Together, these traits improve local microhabitat humidity (He, 2014). Such moisture conditions may further promote the growth of certain aerobic or facultatively anaerobic microorganisms, including Ascomycota. In contrast, the relative abundances of Basidiomycota and Mortierellomycota were lower in the central region but higher in the southern and northern areas. This pattern is likely related to region-specific soil environmental conditions. This study revealed that the latitudinal distribution of Basidiomycota was consistent with soil total nitrogen (TN) content. This correlation may be explained by two factors. First, certain Basidiomycota taxa can decompose organic matter and mediate nitrogen transformation. Increased soil nitrogen availability may therefore indirectly stimulate their growth (Sun et al., 2024). Second, in the central region, vigorous growth of *Z. jujuba* shrublands reduced litter quality, such as lower nitrogen content. This likely limited the decomposition efficiency of saprotrophic Basidiomycota. This shift may have favored more stress-tolerant microorganisms, such as some Ascomycota or bacterial taxa (Aguirre et al., 2024). These groups may have competitively restricted the ecological niche of Basidiomycota. For Mortierellomycota, their abundance showed temperature-dependent variation along the latitudinal gradient. As latitude increased, declining temperatures reduced their reproductive capacity. At the same time, decreased precipitation, drier air, and seasonal temperature fluctuations contributed to their lowest abundance in the central region. Although the northernmost sites had colder climates, the higher soil carbon and nitrogen content there provided compensatory conditions supporting Mortierellomycota survival and proliferation (Zhang et al., 2020).

### 4.2 Response of soil microbial community diversity to latitudinal variation

According to Tables 3, 4, the alpha diversity of the five soil bacterial communities was generally higher than that of the fungal community. This difference may be explained by the faster reproduction rate of bacteria compared with fungi. Bacteria can accumulate diversity more rapidly and show strong adaptability to extreme environments (Fierer and Jackson, 2006; Strickland et al., 2008). Contrary to previous findings (Shen et al., 2023; Duan et al., 2022; Bahram et al., 2018; van den Hoogen et al., 2019), our results revealed a nonlinear trend in most bacterial alpha diversity indices along the latitudinal gradient. These indices initially increased, then decreased, and finally increased again. This pattern may be associated with regional climatic factors. Higher temperatures can alter interspecific competition and resource-use efficiency (Wu et al., 2022). This may reduce microbial diversity in the southern Taihang Mountains. As latitude increases, temperature-adapted species may expand in the central region. This expansion may temporarily enhance microbial diversity. However, with further increases in latitude, bacterial alpha diversity begins to decline, likely due to variations in environmental adaptability among bacterial taxa. In the northernmost regions, cold-tolerant or highly adaptable groups become dominant. Their dominance may contribute to a recovery in diversity (Fierer and Jackson, 2006; Chu et al., 2010; Wu et al., 2011).

For fungal alpha diversity, no consistent latitudinal pattern was observed in our study. This finding differs from previous studies that reported a decrease in fungal diversity with increasing latitude (Bahram et al., 2018; van den Hoogen et al., 2019), However, it supports O′Malley’s proposition of a “stochastic global distribution of microbial communities” (O’Malley, 2007). The peak values of fungal diversity indices, including ACE, Chao1, and Shannon, were mainly observed in the central Taihang Mountains. This spatial pattern may be related to the weakly alkaline soil pH in this region. The pH ranged from 6.5 to 7.5 and appears favorable for maintaining diverse fungal communities (Preem et al., 2021). Both increases and decreases in soil pH can alter nutrient availability. These changes can modify microbial community structure. As a result, microbial diversity may also be affected (Zhou and Ma, 2013). Additionally, well-developed *Z. jujuba* shrublands are present in the central region. These shrubs show vigorous growth and extensive root systems. They provide favorable microhabitat conditions for microbial communities.

Principal Coordinates Analysis (PCoA) revealed that soil bacterial and fungal community structures showed no significant differences among sampling sites. This result is consistent with the global topsoil microbiome study by Bahram et al. (2018). That study showed that bacterial community structure is mainly driven by environmental factors, such as pH, nutrients, and precipitation. It also indicated that geographic distance and latitude play a smaller role. Previous studies have reported that fungal communities tend to converge in areas with similar vegetation types. This pattern may be related to the distribution of *Z. jujuba* in the study region. Permutational multivariate analysis of variance (PERMANOVA) further confirmed the effects of environmental variables. Climatic factors and soil nutrient conditions significantly influenced the *β*-diversity of both bacterial and fungal communities. These findings are consistent with previous studies (Fierer and Jackson, 2006; Tedersoo et al., 2014; Chu et al., 2010). Those studies reported clear shifts in soil bacterial and fungal community structures along latitudinal gradients.

Overall, differences in latitude lead to variation in soil physicochemical properties and climatic conditions. These environmental differences subsequently alter soil microbial community structure.

### 4.3 Effects of environmental factors on soil microbial communities across different latitudinal gradients

Research has shown that climatic factors (Ran et al., 2023) and soil physicochemical properties (Edwards et al., 2015; Xun et al., 2016; Li et al., 2018) drive changes in microbial community structure and diversity. Spearman correlation analysis and Redundancy Analysis (RDA) indicated that climate, soil physicochemical properties, and plant nutrients influenced the distribution of soil microbial communities along the latitudinal gradient. This result is consistent with previous studies (Chang et al., 2025; Cui et al., 2019; Wang et al., 2024).

Abundant nitrogen and soil organic carbon can support higher biomass and greater diversity. Changes in their concentrations may directly affect the abundance and activity of soil bacteria (Liang et al., 2024). However, excessive total soil nitrogen content may inhibit the growth of soil actinomycetes (Qiao et al., 2023). Soil electrical conductivity reflects soil salinity levels, and excessively high salinity can impair the activity of both bacterial and fungal communities (Zhao et al., 2008). This may reduce the relative abundance of microorganisms such as Ascomycota and Basidiomycota. Mean annual precipitation (MAP) influences soil microorganisms mainly by regulating soil moisture and nutrient cycling. These changes affect microbial distribution and community composition. Heavy rainfall can alter decomposition and mineralization rates mediated by soil microbes (Wang et al., 2022; Feng et al., 2018). This process may reshape microbial community structure and metabolic activity. The relative abundance of Acidobacteriota and Basidiomycota tends to increase with higher MAP. This pattern may reflect the preference of some Acidobacteriota subgroups for humid environments. Increased precipitation maintains higher soil moisture and promotes their metabolic activity. Greater rainfall may also lower soil pH. This creates more acidic conditions. Acidobacteriota and Basidiomycota are well adapted to such environments due to their acidophilic characteristics (Jones et al., 2009; Rineau et al., 2012).

The growth density and condition of *Z. jujuba* shrublands varied along the latitudinal gradient. This variation led to differences in soil nutrient inputs. These differences further influenced soil microbial communities. Regression analyses of dominant microbial taxa, diversity indices, and aboveground shrub nutrient content were conducted along the latitudinal gradient (Figures 2, 4). The results showed that *Z. jujuba* shrubs grew most vigorously in the central region of the western Taihang Mountains. This region also exhibited the strongest responses of different microbial groups to environmental factors.

This study used βNTI analysis, null models, and the Neutral Community Model (NCM) to examine microbial community assembly. The results showed that stochastic processes dominated the assembly of both bacterial and fungal communities. However, significant differences existed in the assembly mechanisms between the two microbial groups. These findings are consistent with general patterns of microbial community assembly. They also highlight the uniqueness of northern semi-arid shrub ecosystems. The dominance of stochastic processes agrees with previous studies conducted in the Lvliang region (Li et al., 2024). Those studies reported that fungal communities in shrublands and forests were mainly driven by stochastic processes. This result also aligns with findings from deep desert soils (Sun et al., 2023). In that study, stochastic processes were identified as the core driver of bacterial community assembly. These results suggest that stochastic processes play a broadly consistent regulatory role in soil microbial community assembly. This pattern may be common in shrub ecosystems across northern arid and semi-arid regions.

The weak selective influence of environmental factors further supports the dominance of stochastic processes. The proportion of stochastic processes fluctuated along the latitudinal gradient. At the northern ZL site, heterogeneous selection (HeS) accounted for 80% of the assembly processes. This pattern suggests that climatic differences associated with latitude may enhance deterministic processes at the local scale. For example, variation in mean annual precipitation (MAP) may strengthen environmental selection. This interpretation is consistent with previous studies (Bai et al., 2025). Those studies showed that MAP in desert soils can regulate the shift of microbial assembly processes from stochastic to deterministic mechanisms.

Overall, these findings support the applicability of neutral theory in northern semi-arid shrub ecosystems. They also provide a mechanistic explanation for regional-scale biogeographic patterns of microbial communities.

## 5 Conclusion

The soil microbial communities in *Z. jujuba* shrublands of the Taihang Mountains exhibit distinct latitudinal distribution patterns, revealing the nonlinear response characteristics of the shrublands soil system to climate change, identifying fungi as the core indicator group for the system’s climate sensitivity, clarifying the system stability mechanism dominated by stochastic processes, and deciphering the intrinsic logic of the differential regulation between soil and climate. These findings provide an irreplaceable scientific basis for accurately predicting the impacts of climate change on shrublands ecosystems and formulating targeted soil ecological protection and restoration strategies.

## Data Availability

The original contributions presented in the study are publicly available. This data can be found here: https://www.ncbi.nlm.nih.gov/search/all/?term=PRJNA1275839.
