# Supplementary material for: Latitude distribution characteristics of soil microbial communities in the Ziziphus jujuba var. spinosa shrublands on the western slope of Taihang Mountains
Source: Front Microbiol. 2026 Apr 10;17:1729146. doi: 10.3389/fmicb.2026.1729146 (PMC13106373; doi:10.3389/fmicb.2026.1729146)
Supplement: Supplementary file 1 [file Data_Sheet_1.docx]

| 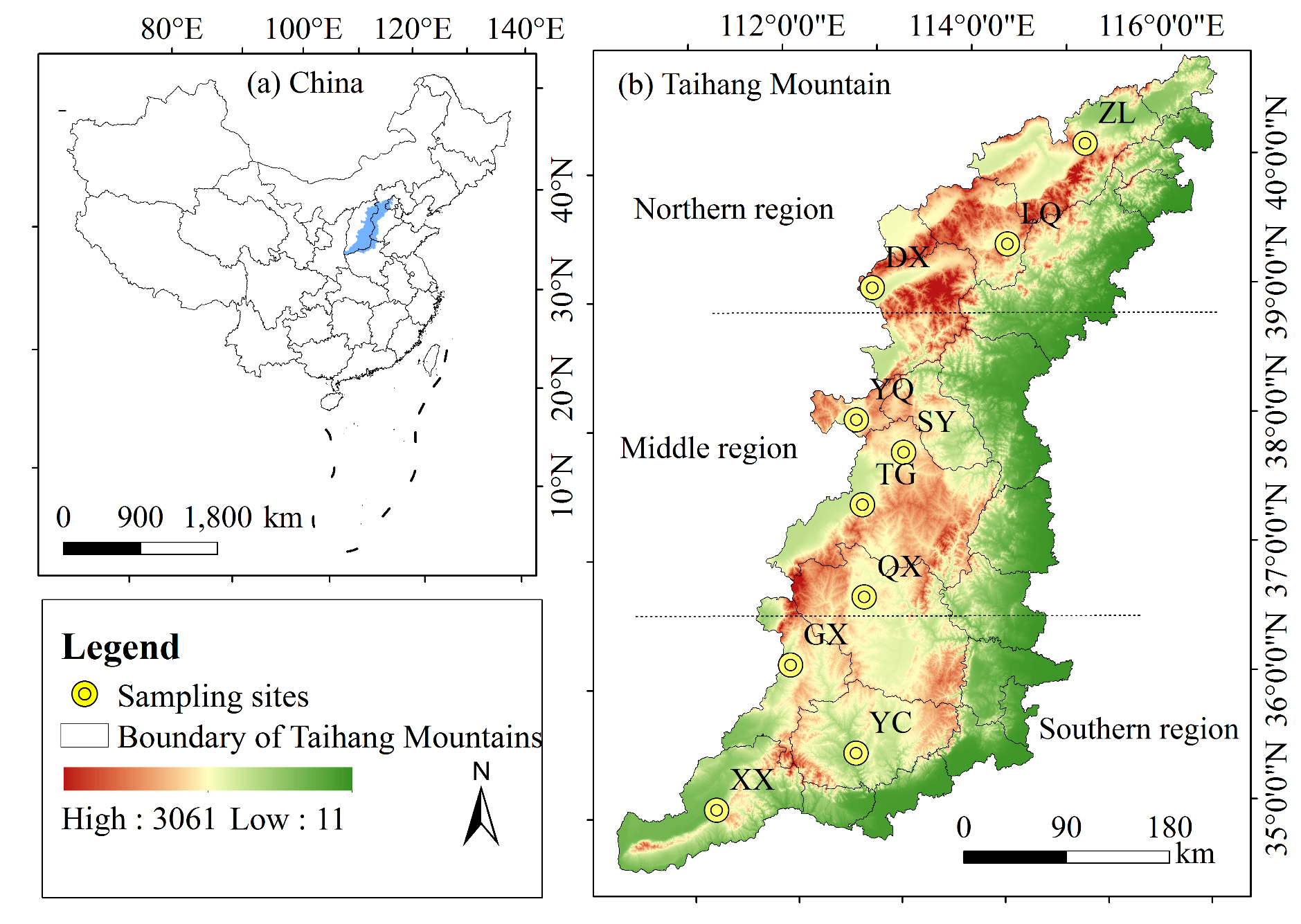 |
| --- |
| **Appendix Fig. 1.** Location Map of the Study Sites  Note: XX refers to Xia County, Yuncheng City, Shanxi Province; YC refers to Yangcheng County, Jincheng City, Shanxi Province; GX refers to Gu County, Linfen City, Shanxi Province; QX refers to Qin County, Changzhi City, Shanxi Province; TG refers to Taigu County, Jinzhong City, Shanxi Province; SY refers to Shouyang County, Jinzhong City, Shanxi Province; YQ refers to Yangqu County, Taiyuan City, Shanxi Province; DX refers to Daixian County, Xinzhou City, Shanxi Province; LQ refers to Lingqiu County, Datong City, Shanxi Province; ZL refers to Zhuolu County, Shijiazhuang City, Hebei Province. |

| 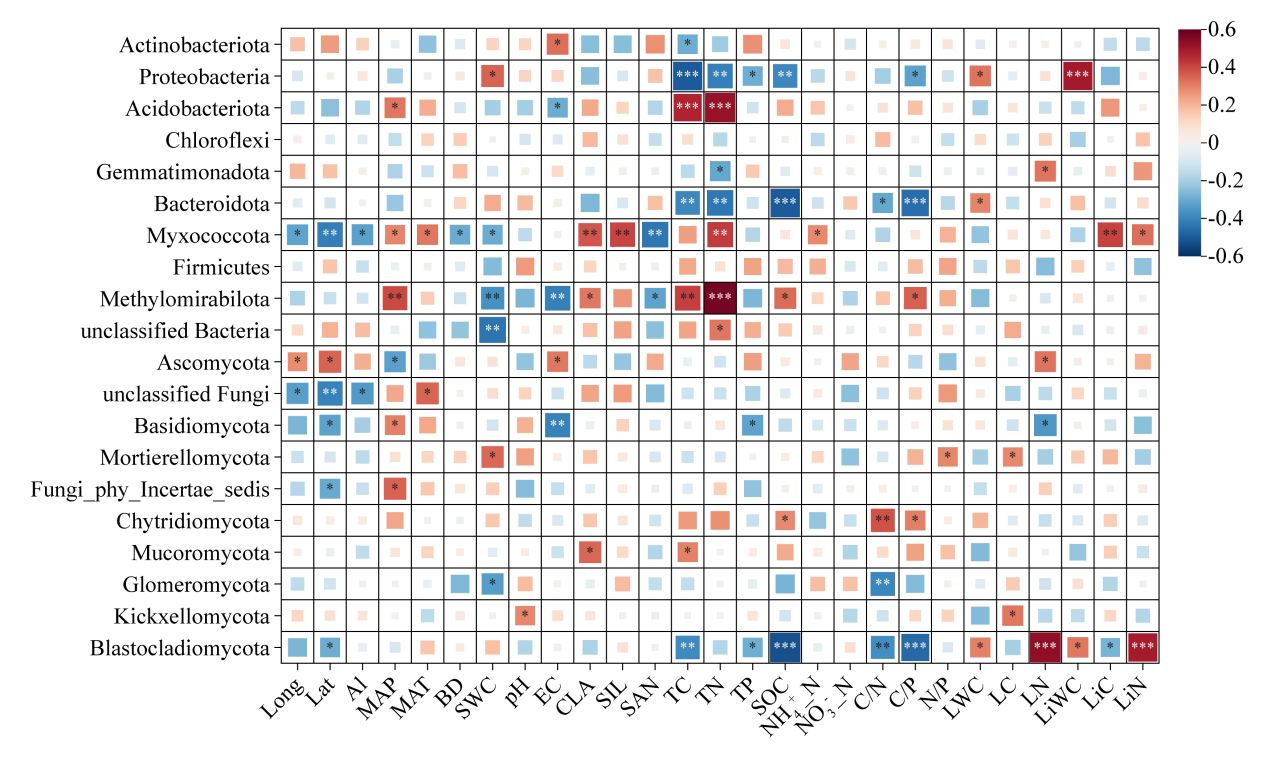 |
| --- |
| **Appendix Fig. 2.** Correlation Analysis Between Different Environmental Factors and Microbial Community Composition |
| Note: Long: Longitude; Lat: Latitude; Al: Altitude; MAP: Mean annual precipitation; MAT: Mean annual temperature; BD: Soil bulk density; SWC: Soil water content; pH: Soil acidity and alkalinity; EC: Soil electrical conductivity; CLA: Soil clay particles; SIL: Soil powder particles; SAN: Soil sand particles; TC: Soil total carbon; TN: Soil total nitrogen; TP: Soil total phosphorus; SOC: Soil organic carbon; NH_4_^+^-N: Ammonium nitrogen; NO_3_^—^-N: Nitrate nitrogen; C/N: Soil carbon to nitrogen ratio; C/P: Soil carbon to phosphorus ratio; N/P: Soil nitrogen to phosphorus ratio; LWC: Leaf water content; LC: Leaf carbon content; LN: Leaf nitrogen content; LiWC: Litter water content; LiC: Litter carbon content; LiN: Litter nitrogen content. |
